# A Twin Study of Altered White Matter Heritability in Youth With Autism Spectrum Disorder

**DOI:** 10.1016/j.jaac.2023.05.030

**Published:** 2023-07-03

**Authors:** John P. Hegarty, Julio C. Monterrey, Qiyuan Tian, Sue C. Cleveland, Xinyi Gong, Jennifer M. Phillips, Olga N. Wolke, Jennifer A. McNab, Joachim F. Hallmayer, Allan L. Reiss, Antonio Y. Hardan, Laura C. Lazzeroni

**Affiliations:** Stanford University School of Medicine, Stanford, California.; Stanford University School of Medicine, Stanford, California.; Tsinghua University School of Medicine, Beijing, China.; Stanford University School of Medicine, Stanford, California.; Stanford University School of Medicine, Stanford, California.; Stanford University School of Medicine, Stanford, California.; Stanford University School of Medicine, Stanford, California.; Stanford University School of Medicine, Stanford, California.; Stanford University School of Medicine, Stanford, California.; Stanford University School of Medicine, Stanford, California.; Stanford University School of Medicine, Stanford, California.; Stanford University School of Medicine, Stanford, California.

**Keywords:** diffusion tensor imaging (DTI), fractional anisotropy (FA), genetic, environment, twin pair difference scores

## Abstract

**Objective:**

White matter alterations are frequently reported in autism spectrum disorder (ASD), yet the etiology is currently unknown. The objective of this investigation was to examine, for the first time, the impact of genetic and environmental factors on white matter microstructure in twins with ASD compared to control twins without ASD.

**Method:**

Diffusion-weighted MRIs were obtained from same-sex twin pairs (6–15 years of age) in which at least 1 twin was diagnosed with ASD or neither twin exhibited a history of neurological or psychiatric disorders. Fractional anisotropy (FA) and mean diffusivity (MD) were examined across different white matter tracts in the brain, and statistical and twin modeling were completed to assess the proportion of variation associated with additive genetic (A) and common/shared (C) or unique (E) environmental factors. We also developed a novel Twin-Pair Difference Score analysis method that produces quantitative estimates of the genetic and environmental contributions to shared covariance between different brain and behavioral traits.

**Results:**

Good-quality data were available from 84 twin pairs, 50 ASD pairs (32 concordant for ASD [16 monozygotic; 16 dizygotic], 16 discordant for ASD [3 monozygotic; 13 dizygotic], and 2 pairs in which 1 twin had ASD and the other exhibited some subthreshold symptoms [1 monozygotic; 1 dizygotic]) and 34 control pairs (20 monozygotic; 14 dizygotic). Average FA and MD across the brain, respectively, were primarily genetically mediated in both control twins (A = 0.80, 95% CI [0.57, 1.02]; A = 0.80 [0.55, 1.04]) and twins concordant for having ASD (A = 0.71 [0.33, 1.09]; A = 0.84 [0.32,1.36]). However, there were also signi primarily associated with differences in general cognitive abilities and perhaps some diagnostic differences for ASD because Twin-Pair Difference-Score analysis indicated that genetic factors may have contributed to ~40% to 50% of the covariation between IQ scores and FA of the corpus callosum. Conversely, the increased impact of environmental factors on some projection and association fibers were primarily associated with differences in symptom severity in twins with ASD; for example, our analyses suggested that unique environmental factors may have contributed to ~10% to 20% of the covariation between autism-related symptom severity and FA of the cerebellar peduncles and external capsule.

**Conclusion:**

White matter alterations in youth with ASD are associated with both genetic contributions and potentially increased vulnerability or responsivity to environmental influences.

**Diversity & Inclusion Statement:**

We worked to ensure sex and gender balance in the recruitment of human participants. We worked to ensure race, ethnic, and/or other types of diversity in the recruitment of human participants. We worked to ensure that the study questionnaires were prepared in an inclusive way. One or more of the authors of this paper self-identifies as a member of one or more historically underrepresented racial and/or ethnic groups in science. One or more of the authors of this paper self-identifies as a member of one or more historically underrepresented sexual and/or gender groups in science. One or more of the authors of this paper self-identifies as living with a disability. The author list of this paper includes contributors from the location and/or community where the research was conducted and they participated in the data collection, design, analysis, and/or interpretation of the work.

During critical periods in perinatal development, molecular cues guide axonal growth cones to target areas in the brain, oligodendrocytes insulate these axons, and synaptic junctions form between neurons that are strengthened or pruned in an activity-dependent manner. These complex and dynamic interactions build the bundles of axons that serve as the primary pathways for information conduction in the central nervous system and support the development of different physiological, behavioral, and cognitive processes. Given their essential role, it should come as no surprise that alterations in the formation and maturation of these white matter tracts are implicated in the pathophysiology of neurodevelopmental disorders, including autism spectrum disorder (ASD). ASD is characterized by difficulties in social communication as well as the presentation of restricted interests and repetitive behaviors early in life, with a clinical phenotype that is extremely heterogenous across individuals. Although the neurobiology of ASD is not yet known, differences in white matter properties in the brain have been reported across post mortem,^1^ clinical neuroimaging,^2^ basic science stem cell,^3^ and translational animal model^4^ studies. Much like the clinical phenotype, white matter alterations are also highly variable across individuals.^5^ Thus, identifying the factors that contribute to variation in white matter in the brain in youth with ASD is a critical step for advancing our knowledge regarding the underlying pathophysiology of the disorder.

Twin studies have been instrumental in teasing apart the role of genetic vs environmental factors that contribute to autism-related variation. Twin studies were critical in establishing the heritability of ASD,^6,7^ with large-scale association studies reporting that approximately 10% to 25% of cases are associated with various rare genetic abnormalities, whereas the remaining cases most likely originate from a combination of multiple interacting genetic variants (ie, polygenic) and a range of genetic modifiers. In addition to a preponderance of evidence for the genetic basis of autism, twin studies also report that environmental factors may play an important role,^8,9^ including our own research, which found a higher association between environmental factors and morphometric properties of the brain in twins with ASD compared to control twins.^10–12^ This preliminary evidence suggests that although both genetic and environmental factors contribute to autism-related variation in the brain,^11–13^ there may be increased environmental influences on some regional white matter properties in twins with ASD.^12^ Yet, to date, no twin studies have examined these relationships using gold-standard diffusion tensor imaging (DTI). DTI calculates the rate of water diffusion per voxel in the brain,^14^ and these data can be used to infer differences in tissue architecture. There are several diffusion rates that can be calculated, of which fractional anisotropy (FA) and mean diffusivity (MD) are the most common. FA is the relative directional preference of diffusion in each voxel, whereas MD is simply the average length of diffusion vectors. These values are used as metrics for the integrity of white matter tracts in the brain and are consistently altered in autistic people compared to people without ASD.^5,15^ Overall, it appears that alterations in the integrity of white matter tracts in the brain may be involved in the pathophysiology of ASD, but the impact of genetic and environmental factors on white matter development has not yet been investigated in a clinical sample using the twin model design.

The objectives of the current investigation were as follows: to compare white matter properties in the brain between twins with ASD and control twins without any neurological or psychiatric diagnoses; to examine the proportion of variation in FA and MD that are associated with additive genetic (A) and common/shared (C) and unique/unshared (E) environmental factors; and to explore the specificity of these effects across the brain. Based on previous research, we hypothesized that commissural fibers like the corpus callosum,^5,15^ as well as projection fibers like the cerebellar peduncles,^16^ would exhibit group-related differences in FA and MD between twins with ASD and control twins. We also predicted that FA and, to a lesser extent, MD would be primarily genetically mediated in the majority of tracts across the brain in control twins, and that environmental influences would be predominantly limited to limbic fibers such as the fornix as well as some projection fibers such as the corticospinal tract.^17–21^ Conversely, we expected that autistic twins would exhibit a larger proportion of variation in white matter properties attributed to environmental factors, especially in the cerebellar peduncles, because they exhibited increased environmental associations in our preliminary examination of independent T1-weighted images from this sample^12^ and may be especially vulnerable to the early environment in ASD.^22^

## METHOD

### Participants

Inclusion criteria were same-sex twin pairs between the ages of 3 and 14 years at the time of enrollment. For twin pairs with ASD, 1 or both twins were required to have an ASD diagnosis (ie, discordant or concordant for ASD, respectively) and to be testable on standardized measures of ASD symptoms and cognitive abilities. For control twin pairs, both twins were required to have a birth weight of at least 1,500 g and a history of regular school attendance and performance consistent with the appropriate developmental level.

Exclusion criteria were as follows: pregnancy, a diagnosis of an active neurological disorder (eg, epilepsy) or significant medical problem (eg, diabetes), or the inability to undergo magnetic resonance imging (MRI) scanning (eg, ferrous metal implant). For twin pairs with ASD, 1 or both twins could not be diagnosed with another clinically significant psychiatric disorder (eg, schizophrenia, bipolar disorder). For control twin pairs, neither twin could have a diagnosis of ASD or a history of a psychiatric disorder, including any behaviors above threshold for behavioral or emotional problems on standardized assessment measures (ie, the Child Behavior Checklist^23^).

Study enrollment and primary data collection were conducted between 2010 and 2015. Initially, potential twin pair participants were identified from the California Autism Twin Study^9^ (27%) and Interactive Autism Network Research Database (33%). The remaining twin pairs were recruited from online advertisements and direct outreach in the local and greater autism community. Demographic information and medical history were collected using custom surveys to support screening procedures. Socio-economic status was calculated using the Hollingshead method, which incorporates family structure, employment status and field, and educational attainment into a single score (A.A. Hollingshead, unpublished data, 1975). Following screening, 90 same-sex twin pairs (N = 180) were invited to participate, 55 in which at least 1 twin was diagnosed with ASD and 35 in which neither twin was diagnosed with ASD. DNA from saliva samples for both twins were used to confirm zygosity (ie, monozygotic [MZ] or dizygotic [DZ] for each twin pair).^9^ The methodology of this investigation was approved by our Institutional Review Board, and written consent was obtained from parents/caregivers and assent from participants after discussion of the study procedures. Additional information on the study design was reported in previous examinations of the morphometric and neurochemical properties from separate MRI scans that were collected from this sample.^10–12,24^

### Cognitive and Behavioral Testing

Clinical diagnoses of ASD were confirmed using the Autism Diagnostic Observation Schedule, 2nd Edition,^25^ a direct observation of the child’s behaviors, and the Autism Diagnostic InterviewRevised,^26^ a structured parent interview regarding developmental history. The Social Responsiveness Scale (SRS),^27^ a checklist of autism-related symptoms in social communication and restricted and repetitive behaviors, was also obtained to compare core ASD symptoms between groups. The SRS was designed to provide a continuous measure of autistic symptoms across a continuum from the general population to those diagnosed with ASD. The Stanford–Binet Intelligence Scales, 5th Edition,^28^ was used to assess general cognitive abilities and to generate intelligence quotient (IQ) scores.

### Magnetic Resonance Imaging

**FIGURE 1:**
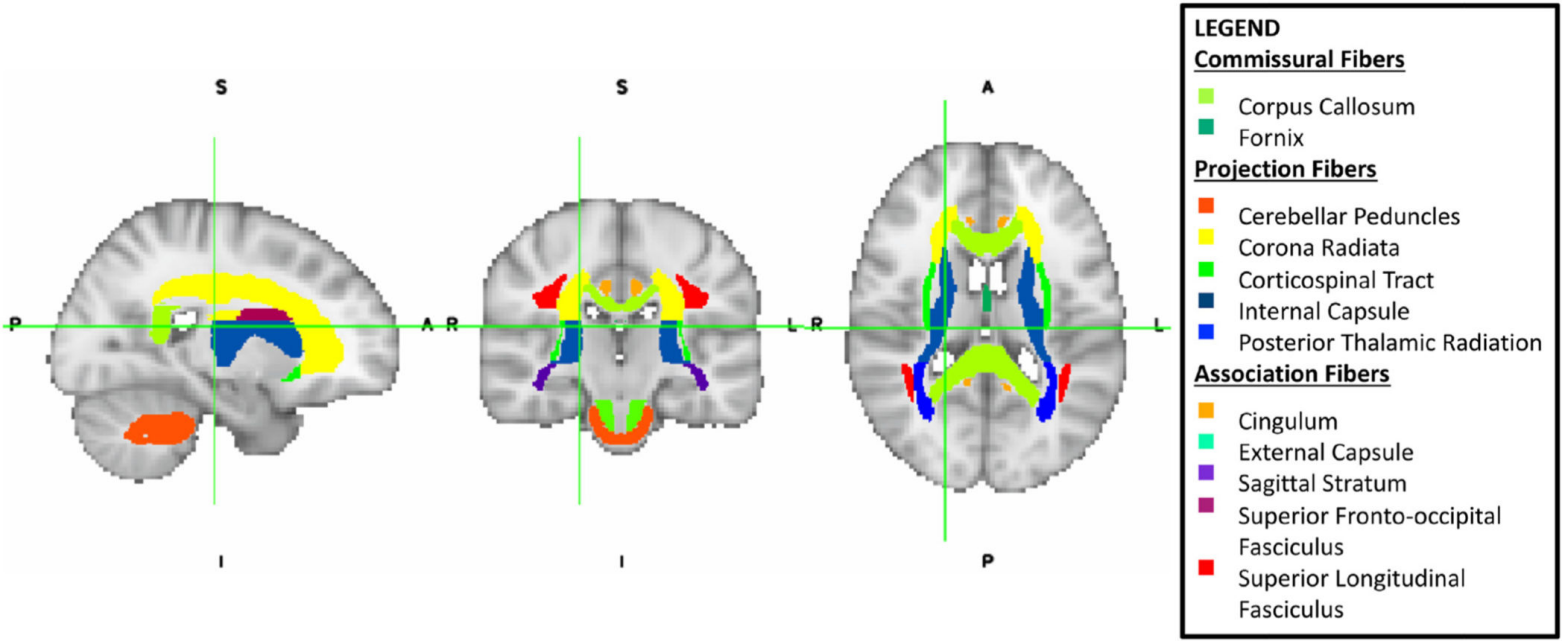
White Matter Tracts

Neuroimaging was conducted on General Electric 3T MR750 scanners (Waukesha, WI) using standard 8-channel head coils. All participants were assessed on an MRI simulator prior to data collection, and any participants who were not within acceptable motion thresholds were provided additional training, offered the use of light procedural sedation, or excluded from the study. Participants with autism who were unable to remain motionless for scanning were administered light procedural sedation with propofol at a rate of 200 to 300 μg/kg per minute under the supervision of an anesthesiologist. Four separate diffusion-weighted product sequences were acquired from each participant with the following parameters: field of view = 24 cm, matrix size = 128 × 128, echo time = minimum, response time = 5,700 msec, 45 axial slices, slice thickness = 2.9 mm, skip = 0. A total of 25 diffusion directions were acquired with diffusion weighting b = 1,000 s/mm^2^. Processing procedures were conducted using NiftyReg^29^ and the FMRIB Software Library Diffusion Toolbox using Tract-Based Spatial Statistics.^30^ Trained raters visually inspected all images before inclusion, and then the diffusion data within each participant were co-registered. To compensate for the minor residual nonlinear deformation, b0 images from the different repetitions were non-linearly co-registered and the resultant non-linear transformations were applied to the diffusion data. The diffusion encoding directions were then rotated based on the obtained affine transformations, and the coregistered repetitions were combined. Next, the combined diffusion data were corrected for eddy current distortions and bulk motion, and diffusion tensor model fitting was performed to derive the FA map and the voxel-wise primary diffusion orientations. FA, which ranges from a value of 0 (isotropic) to 1 (anisotropic), and MD provide measures of complementary properties of white matter microstructure and were the primary metrics of white matter integrity that were examined in this study. Images were segmented into independent tracts from the John Hopkins University white matter atlas, as outlined in the ENIGMA protocol reported by Jahanshad *et al*.^21^ (Figure 1). This approach allowed the assessment of genetic and environmental influences on white matter properties across the entire brain, while minimizing the number of regions of interest for multiple comparisons, and also supported direct comparison to heritability estimates from previous studies of twin pairs without ASD. Based on our preliminary findings,^12^ we also added the cerebellar peduncles, as this was an *a priori* region of interest. FA and MD were then calculated as averages across all voxels within each tract. Additional information on our image processing and analysis pipeline is outlined in Supplement 1, available online.

### Statistical Analyses

Group matching was examined with independent- and paired-samples *t* tests and χ^2^ tests using IBM SPSS Statistics. Between-group comparisons (ASD vs control) of DTI measures were completed with linear mixed modeling using SAS, accommodating for the fixed effects of age and sex and random effects of “family” (ie, within twin pairs) and “genetics” (ie, MZ vs DZ). From twin pairs discordant for ASD, twins with ASD were included in group-matching analyses, whereas twins without ASD were excluded. Within-group comparisons of the discordant twin pairs (ASD vs co-twin) were also examined using repeated-measures structured covariance models. False discovery rate (FDR)^31^ correction was used to account for multiple comparisons.

Prior to conducting the formal twin analyses, the reliability of the model’s assumptions were assessed using intraclass correlations (ICC), adjusted for sex and diagnosis, for all twin pairs, including those concordant and discordant for having ASD. Separate ICCs were also examined for twin pairs that were concordant for having ASD and controls pairs that were concordant for not having ASD to compare the magnitude of the within-pair correlations between the MZ and DZ pairs in the different participant groups. This comparison provides an initial assessment of whether there are salient differences that contribute to the twin modeling results. Next, twin modeling was carried out using 2 distinct types of analyses for this study.

#### Classical ACE Twin Model Analyses.

The first type of twin analysis fit the classical ACE variance–component twin model, which traditionally estimates the proportion of trait variance (PTV) attributable to each of 3 sources: (A) additive genetic effects; (C) common environmental effects preferentially shared by both members of a twin pair; and (E) unique environmental effects unshared by both members of a twin pair. To fit the classical ACE twin model, we used generalized DeFries–Fulker (DF) regression, described in detail elsewhere,^32,33^ and implemented it using STATA. Generalized DF regression uses an iterative regression model applied to MZ and DZ twin data to fit the ACE model in an unbiased way. Bootstrap analysis with 1,000 replications was used to compute statistical significance and CI. When A or C was initially non-significant in the model, we applied sequential elimination and fit the more parsimonious AE or CE model instead.

#### Twin-Pair Difference-Score Analyses.

The second type of twin analysis explores the putative genetic or environmental pathways underlying a pair of shared traits or phenotypes X and Y, which, in this case, comprise 1 brain measure (eg, FA in the corpus callosum), and 1 behavioral measure (eg, social communication impairments on the SRS). This novel method uses twin-pair difference-scores (TPDSs) to extend and modify the “MZ difference score” method first proposed by Pike *et al*.,^34^ which was an application to MZ twins of the classic statistical matched-pair design that has since been used by a number of authors, including ourselves.^11,35–38^ To our knowledge, we present the first formal mathematical rationale for difference scores in the twin model context. The resulting novel TPDS method adds to the original MZ difference score method in 3 ways: it (1) incorporates information from DZ twins; (2) relies on covariances instead of correlations, and as a consequence; (3) provides quantitative estimates of the contributions to pathway covariance (CPCs) from both additive genetic (A) and unique or unshared (E) environmental factors. Thus, TPDSs enable a comparative evaluation of the role of genetic and unshared environmental pathways within and between different pairs of traits.

In Supplement 1, available online, we present a full description and proof for the novel TPDS method. We consider the decomposition of trait X as b_1_ A + b_2_ C + b_3_ E + b_4_ U, where A, C, and E encompass all shared latent factors or variables in the pathway connecting X and Y. Specifically, A represents genetic variants acting in an additive way; C represents environmental variables shared in common by co-twins; and E represents environmental variables not shared by co-twins. U represents variables contributing to trait X, but not Y. The b’s are regression coefficients. We use a similar decomposition of trait Y, but with regression coefficients labelled with d’s. Let V(A) and V(E) refer to the variances of A and E, respectively. Then, the additive X-Y genetic CPC equals b_1_d_1_V(A) and the non-shared environmental X-Y CPC equals b_3_ d_3_V(E). Let the covariance of the difference scores in MZ and DZ twins be, respectively, Cov_MZ_(X_1_–X_2_, Y_1_–Y_2_ ) and Cov_DZ_(X_1_–X_2_, Y_1_–Y_2_ ). In Supplement 1, we demonstrate that the additive genetic CPC can be estimated as Cov_MZ_(X_1_–X_2_, Y_1_–Y_2_ ) Cov_DZ_(X_1_–X_2_, Y_1_–Y_2_ ) and the non-shared environmental CPC can be estimated by Cov_MZ_(X_1_–X_2_, Y_1_–Y_2_ ) / 2. Genetic and environmental CPCs may be positive or negative, and may have opposite signs if they act to offset each other. A significant non-zero covariance between the MZ difference scores of 2 traits can be interpreted to mean that both traits are associated with at least some of the same latent environmental factors. Significant inequality of MZ and DZ difference score covariances can be interpreted to mean that both traits are associated with at least some of the same unobserved genetic factors. One cannot infer the direction of causality from this model alone.

#### TPDS Brain–Behavior Analyses.

To explore the influence of genetic and environmental factors on important brain–behavior relationships, we used TPDS analysis as outlined in Supplement 1 to white matter properties and symptom severity, as measured by the SRS,^27^ and general cognitive abilities, as measured by the Stanford–Binet Intelligence Scales, 5th Edition.^28^ First, we standardized brain and symptom severity variables within individuals to have mean 0 and variance 1. We then calculated TPDSs for scores on subscales of the SRS (total, social communication impairments [SCI], and autistic mannerisms/restricted, repetitive behaviors [RRB]) and the Stanford–Binet (full-scale IQ [FSIQ], verbal IQ [VIQ], and non-verbal IQ [NVIQ]) with FA from the whole brain and 12 white matter tracts, with covariances of the TPDSs generated separately for MZ and DZ twin pairs. See Supplement 1, available online, for percentile-based 95% CIs generated using the bootstrap with 10,000 replications. Given the hypothesis-generating nature of this TPDS analysis, CIs were not adjusted for multiple comparisons, and only the most robust relationships are discussed below.

## RESULTS

### Participant Characteristics and Demographics

Of the 90 twin pairs who participated, there were 6 from whom we could not obtain scans from both twins. Diffusion-weighted data were available from 84 complete twin pairs (N = 168), 32 concordant for having ASD (16 MZ; 16 DZ), 16 discordant for ASD (3 MZ; 13 DZ), 2 pairs in which 1 twin had ASD and the other exhibited some subthreshold symptoms (1 MZ; 1 DZ), and 34 control pairs (20 MZ; 14 DZ). Groups were well matched, as there were no differences in potentially confounding demographic influences (Table 1). Of note, there was a 95% overlap between the participants included in this DTI sample and participants in the previously reported T1 morphometric study from this twin dataset.^10,11^

### Group Differences in FA and MD

Five twin pairs were excluded from FA analyses because at least 1 twin was identified as an extreme statistical outlier for global FA, leaving 75 twins with ASD, 17 co-twins without ASD from discordant or subthreshold twin pairs, and 66 control twins. As expected, average FA was reduced in twins with ASD compared to controls (mean difference = −0.014, 95% CI [−0.021, −0.008]) (Table 2). Tract-specific comparisons that survived correction for multiple comparisons included some commissural and association fibers, such as the corpus callosum (−0.021 [−0.031, −0.010])] and external capsule (−0.013 [−0.022, −0.004]), respectively; but the vast majority of differences were found in projection fibers, including the cerebellar peduncles (−0.013 [−0.023, −0.003]), corona radiata (−0.009 [−0.017, −0.002]), corticospinal tract (−0.028 [−0.050, −0.009]), internal capsule (−0.013 [−0.021, −0.005]) and posterior thalamic radiation (−0.025 [−0.040, −0.010]). Notably, differences in FA were also found in twin pairs discordant for ASD for the average (−0.012 [−0.022, −0.002]) and corona radiata (−0.023 [−0.04, −0.006]) (Table S1, available online).

Five pairs were also excluded from MD analyses as statistical outliers, leaving 76 twins with ASD, 18 co-twins without ASD from discordant or subthreshold twin pairs, and 64 controls. There were considerably fewer autism-related differences in MD (Table 2). Moderately higher estimates were found in twins with ASD for some association and projection fibers compared to controls, such as the superior longitudinal fasciculus (mean difference = 0.013 × 10^−3^, 95% CI [0.001, 0.025]) and corona radiata (0.023 × 10^−3^ [0.006, 0.040]); but they did not survive correction for multiple comparisons. Similarly, only modest differences were found in twin pairs discordant for ASD, and they did not survive correction for multiple comparisons either (Table S1, available online).

### Intraclass Correlations

**FIGURE 2:**
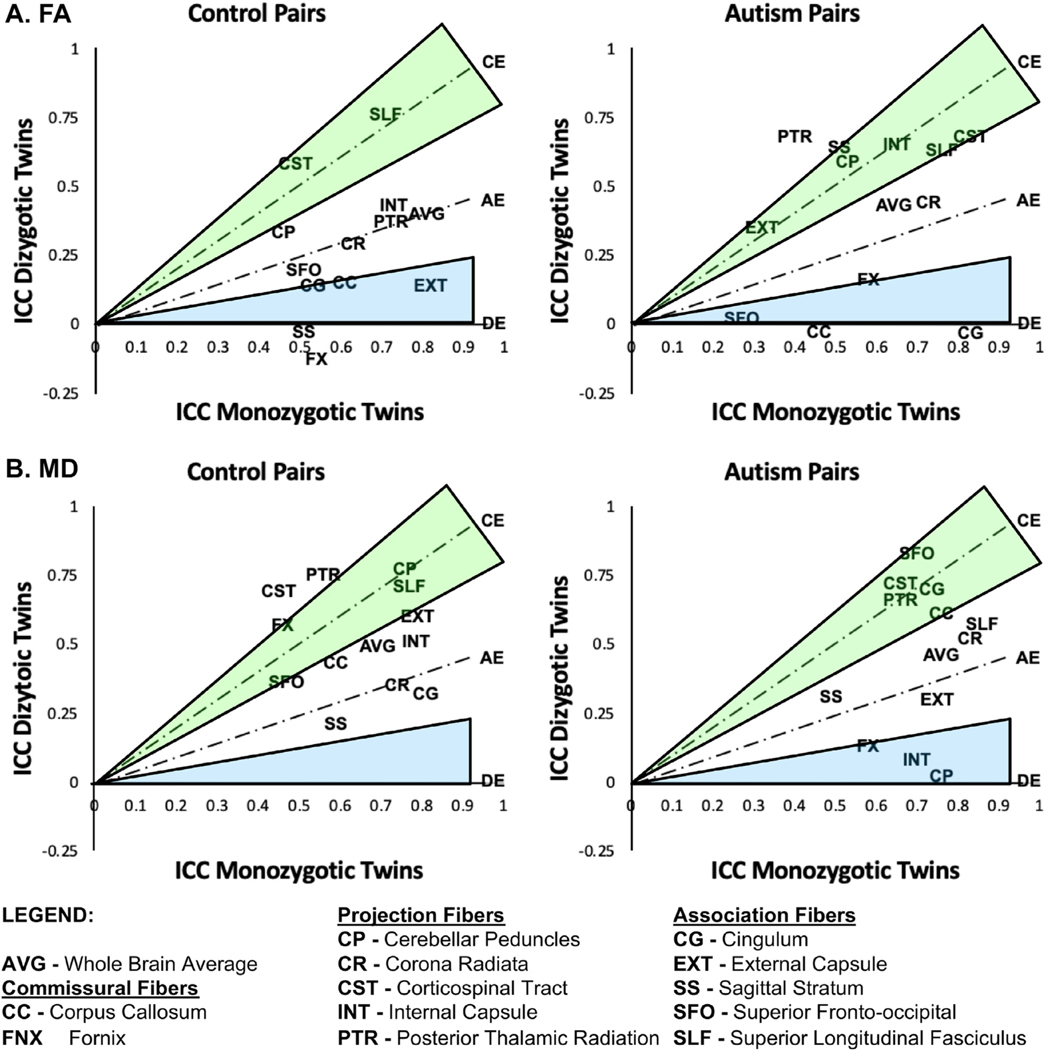
Visual Depiction of the Classical ACE Twin Model **Note:** Intraclass correlations (ICC) of fractional anisotropy (FA) and mean diffusivity (MD), adjusted for sex, were generated separately for monozygotic (MZ) and dizygotic (DZ) twin pairs in which both twins were concordant for having autism or both were controls without autism. ICCs are displayed in relation to ACE model space (A = additive genetics; C = common or shared family environment; E = unique or unshared environment; D = genetic dominance). Tracts near or above the CE line (green) are primarily environmentally mediated; tracts near or around the AE line are approximately equally mediated by genetic and environmental factors; and tracts near or below the DE line (blue) are primarily genetically mediated.

Intraclass correlations (ICC) across all twin pairs, regardless of ASD diagnosis, were generated to examine twin model assumptions for FA and MD. These ICCs generally met the assumptions for twin modeling^39^; for example, they were statistically significant and higher in MZ compared to DZ twin pairs for the majority of tracts (Table S2, available online). Focusing more specifically on twin pairs concordant for having ASD and controls concordant for not having ASD, we found that ICCs for FA in MZ twins were generally of large magnitude in both groups (ICC = 0.40–0.83), excluding some projection fibers in twins with ASD such as the external capsule and superior fronto-occipital fasciculus (Figure 2; Table S3, available online). ICCs were also higher in MZ twins concordant for ASD compared to controls for some association [cingulum (ICC_ASD_ = 0.83; ICC_CTRL_ = 0.54)] and projection fibers [corticospinal tract (ICC_ASD_ = 0.83; ICC_CTRL_ = 0.49)]; yet also lower in others, such as the external capsule (ICC_ASD_ = 0.32; ICC_CTRL_ = 0.82) and posterior thalamic radiation (ICC_ASD_ = 0.40; ICC_CTRL_ = 0.73). The external capsule was the only difference that survived correction for multiple comparisons.

In DZ twin pairs, ICCs were generally of lower magnitude compared to MZ twins in both concordant ASD and control groups, but there were some tracts that exhibited moderately higher ICCs, such as the external capsule, sagittal stratum, cerebellar peduncles, and posterior thalamic radiation in twins concordant for ASD and the superior longitudinal fasciculus and corticospinal tract in control twins. The ICC for the sagittal stratum was also marginally higher in DZ twins concordant for ASD compared to controls (ICC_ASD_ = 0.63; ICC_CTRL_ = −0.04), but this difference did not survive correction for multiple comparisons.

ICCs for MD were also generally of large magnitude for MZ twins concordant for having ASD and control twins concordant for not having ASD (ICC = 0.45–0.86), but there were no group-related differences (Figure 2; Table S3, available online). In DZ twin pairs, ICCs for MD were generally of lower magnitude compared to MZ twins for both groups, except for a few projection (eg, corticospinal tract and posterior thalamic radiation) and association fibers (eg, superior fronto-occipital fasciculus). The fornix ICC was also moderately higher in DZ compared to MZ control twins. ICCs for DZ twins were lower in concordant ASD pairs compared to controls for the cerebellar peduncles (ICC_ASD_ = 0.001; ICC_CTRL_ = 0.76), yet higher for the superior fronto-occipital fasciculus (ICC_ASD_ = 0.83; ICC_CTRL_ = 0.36); both of which survived correction for multiple comparisons.

### Classical ACE Twin Model

ACE modeling provides estimates for the proportion of variance that is associated with additive genetic (A) and common/shared (C) or unique/unshared (E) environmental factors.^39^ Our data were best fit with simplified AE or CE models for all of the measures that were examined (Table 3). Within control pairs, average FA and the majority of commissural, projection, and association fibers were predominantly associated with genetic factors (A = 0.52–0.80), except for the cerebellar peduncles, corticospinal tract, and superior longitudinal fasciculus. Within twin pairs concordant for ASD, average and commissural fiber FA were also predominantly associated with genetic factors, but this extended only to a subset of projection and association fibers. Conversely, twins with ASD exhibited more environmental influences on the majority of their projection and association fibers (C = 0.34–0.77), including the cerebellar peduncles, corticospinal tract, posterior thalamic radiation, internal and external capsules, sagittal stratum, and superior longitudinal fasciculus.

Average MD across the brain was also primarily genetically mediated in both control twins and twins concordant for having ASD (A = 0.80–0.84). This was also the case for the majority of tracts in twin pairs with ASD (A = 0.49–0.94) but not for control twins. Conversely, the majority of tracts in control twins were predominantly environmentally mediated (C = 0.39–0.76), including commissural (eg, corpus callosum), projection (eg, corticospinal tract), and association fibers (eg, superior frontooccipital and longitudinal fasciculi). The only tracts that were primarily environmentally mediated in twins with ASD were the corpus callosum, corona radiata, posterior thalamic radiation, cingulum, and superior fronto-occipital fasciculus (C = 0.64–0.78).

### TPDS Brain–Behavior Analyses

Genetic factors (A) appeared to have the greatest impact on brain–behavior relationships between white matter integrity and general cognitive abilities, ie, full-scale, verbal, and non-verbal IQs (Table S4, available online). These relationships were found in all twin pairs across the entire brain such that ~40% to 50% of the variance in full-scale, verbal, and non-verbal IQs were associated with covariance in average FA as well as for commissural fibers such as the corpus callosum. In addition, similar influences of genetic factors on the relationships between IQ and FA were found for projection fibers such as the corona radiata (54%−63%) and internal capsule (21%−24%) and more specifically for non-verbal IQ with association fibers such as the sagittal stratum (46%) and superior longitudinal fasciculus (31%). Most of these relationships were replicated in control pairs but only for non-verbal IQ (~20%) and not including the association fibers. Relationships between IQ and FA were much more widespread in twins with ASD compared to control twins, especially when including both concordant and discordant pairs. For instance, similar relationships across IQ scores were found for average FA (51%−57%), the corpus callosum (65%−69%), and projection fibers such as the corona radiata (60%−72%) as well as between nonverbal IQ and association fibers such as the superior longitudinal fasciculus (39%).

Genetic factors had much less of an impact on the brain–behavior relationships between white matter integrity and autism-related symptoms from the SRS (Table S5, available online). In all twin pairs, covariance of FA in the external capsule was associated with covariance in total scores on the SRS as well as the SCI and RRB subscales (38%−44%). Most of these relationships were replicated in all ASD twin pairs (58%- 61%) but not control twins, who instead showed minimal relationships between autistic symptoms and FA that were primarily limited to RRB and association fibers such as the superior longitudinal fasciculus (~7%). Twin pairs concordant for ASD also exhibited an even greater impact of genetic factors on the relationships between FA and symptom severity for association fibers such as the superior longitudinal fasciculus, especially for RRB (92%), and superior fronto-occipital fasciculus, especially for SCI (99%), as well as commissural fibers such as the fornix (~71%−86%), although the small sample size should be considered regarding the magnitude of these estimates.

Conversely, unique environmental factors (E) had minimal impact on the relationships between white matter integrity and general cognitive abilities, and instead appeared to have their greatest impact on brain–behavior relationships for autism-related symptom severity (Tables S6 and S7, available online). Across all twin pairs, these relationships were found for projection fibers (8%−10%) such as the cerebellar peduncles, especially for SCI (10%), as well as association fibers such as the external capsule, which were across symptom domains (6%−9%). These relationships were also replicated in examinations of all autistic twins, with 14% to 22% shared covariances in the cerebellar peduncles and 12% to 17% in the external capsule, as well as for examinations of twin pairs concordant for ASD, with 15% to 23% shared covariance for the cerebellar peduncles and 13% to 19% for the external capsule. These relationships stood out in comparison to those of control twins, who exhibited only modest environmental impacts that were limited to the superior fronto-occipital fasciculus(~3%−4%),which could be related to floor effects of this measure for individuals who display only minimal autism-related behaviors.

Environmental factors appeared to have much less of an impact on brain–behavior relationships between white matter integrity and general cognitive abilities as assessed from IQ scores. Across all twin pairs, there was a relationship between covariance in FA of the corpus callosum and verbal IQ (−3%) as well as in control pairs for association fibers such as the sagittal stratum (~ −3% to −5%) and commissural fibers such as the fornix (−4%), but these relationships were modest and not replicated across different sets of twin pair comparisons. It is also important to note that the findings from the quantitative estimates of the novel Twin-Pair Difference-Score method agree very closely with qualitative results from the heuristic difference score approach used in previous investigations,^10,11^ with the new method potentially being more reliable and sensitive to important brain–behavior relationships.

## DISCUSSION

White matter alterations are frequently reported in ASD, and we replicated some of the most consistent findings in this novel twin sample. Twin modeling analyses further indicated that variation in white matter microstructure was predominantly genetically mediated in twins without psychiatric or neurological diagnoses but was substantially more environmentally mediated in twins with ASD, especially regarding FA. Twin-pair differences in FA also covaried with differences in IQ and autism symptom severity, with genetic (A) variation primarily associated with general cognitive abilities and unique environmental (E) variation primarily associated with core symptom severity. Thus, genetic factors associated with ASD may contribute to the development of white matter properties that underlie some differences in general cognitive abilities as well as a diagnosis of the disorder, whereas unique environmental exposures may more directly contribute to the development of white matter properties that mediate some of the extreme heterogeneity in symptom presentation across individuals, which also corroborates our previous findings regarding gray matter properties in twins with ASD.^10^ These results are promising, because they suggest that some treatments targeting the early environment could potentially alter the course of neurodevelopment to provide clinical benefits in the core symptom domains of ASD.

To our knowledge, this is the first investigation to examine diffusion tensor imaging in twins with and without ASD. Our findings point to widespread reductions in FA across the brain, which is consistent with the current literature^5,15^ and further supports that alterations in white matter architecture may underlie some variation in autism-related behaviors. Considering that FA generally increases during brain maturation,^40^ widespread reductions would be a plausible outcome for a neurodevelopmental disorder. In this and previous studies, some of the largest reported differences were in commissural fibers, such as the corpus callosum.^5^ We also observed consistent alterations across projection fibers but only modest differences in association fibers in the current sample. Differences in MD in some association and projection fibers were also found, but they did not survive correction for multiple comparisons. Overall, the observed group-related alterations suggest that our dataset may provide a representative sample to investigate twins with idiopathic forms of ASD, at least as representative as possible for a heterogeneous population defined by subjective and categorical diagnostic thresholds. Our sample of control twins also exhibited patterns of genetic and environmental influences on white matter properties similar to those of previous large-scale twin studies that used a similar approach,^21^ suggesting that our control sample also provided an adequate comparison group. Although the majority of general twin studies indicate that white matter development is heavily influenced by genetic factors,^20,21,41,42^ there is also disagreement across reports^43^ and substantial heterogeneity as to the extent and impact across different tracts and measures, which should be considered when interpreting our findings.

Comparing genetic and environmental influences between the autism and control groups using the classical ACE twin model design, it appeared that twins with ASD exhibited a similar impact of genetic factors on whole-brain and commissural FA to that of twins without psychiatric or neurological diagnoses. This finding, in conjunction with the aforementioned group-related differences, indicate that some genetic contributions to ASD^6,7^ may alter white matter development of inter-hemispheric tracts. In addition, we also observed significant environmental influences on projection fibers, such as the cerebellar peduncles, and association fibers, such as the superior longitudinal fasciculus, in both groups. These cerebellar findings are noteworthy because they corroborate our previous report regarding environmental effects on cerebellar white matter volume in twins with ASD^12^ as well as theories of cerebellar contributions to autism.^22^ The cerebellum may also be particularly vulnerable to environmental influences during critical periods of neurodevelopment.^44^ There were even more widespread environmental influences on some projection and association fibers in twins with ASD compared to controls. The external capsule and posterior thalamic radiation findings are interesting because we previously reported that deep gray matter structures, such as those connected by these tracts, were largely under genetic control in twins with ASD.^10,12^ Thus, these results provide new insights into how both genetic and environmental factors can affect the same brain circuits but differentially impact cognition and behavior. There also appeared to be potentially increased genetic influences on MD in some of these same tracts. This seemingly antithetical relationship mirrors the differential impacts that we previously found between genetic and environmental factors on gray matter surface area vs cortical thickness, as well as their associations with phenotypic variability in ASD. Clearly, these gene–environment interactions have complex impacts on brain development that warrant further investigation, especially regarding their contribution to individual differences in cognition, behavior, and mental health.

This is also the first investigation to describe a formal proof for the use of difference scores in the analysis of twin-pair data and, as a consequence, provide quantitative estimates for comparing genetic and envonmental impacts on trait covariances on important brain–behavior relationships. The method examines trait covariation through twin-pair difference-scores, which provides a means of evaluating whether genetic or unique environmental factors might contribute to the relationship between FA and measures of general cognitive abilities and autism-related symptom severity. Based on comparisons between different sets of twin pairs, it appeared that overall brain–behavior relationships for FA in tracts that were highly heritable were also primarily associated with genetic contributions to covariation in general cognitive abilities.^45^ Similarly, the cognitive and behavioral features that were associated with tracts that were primarily environmentally mediated were also predominantly associated with environmental contributions to core ASD symptoms. This is consistent with our previous report of potential environmental influences on the relationship between gray matter properties and the severity of restricted and repetitive behaviors in twins with ASD,^10^ although there were some shared genetic and environmental influences on tracts such as the external capsule and corpus callosum. Cumulatively, it appears that genetic modulation of white matter development in commissural fibers may contribute to general cognitive abilities and some differences in the diagnosis of ASD, whereas the effects of environmental influences on white matter development in some association and projections fibers may contribute more directly to differences in the presentation and severity of core ASD symptomatology. Albeit the effects of these factors on brain development are much more complex than could be modeled in the current study, an inherent limitation of our approach and the modest twin sample size, this research still provides important preliminary findings regarding the potential pathways through which genetic factors and environmental influences may have an impact on both biological and psychological variation during neurodevelopment.

There are several additional limitations of our research that should be considered. First, our use of the SRS to compare autism symptoms between different individuals may not be able to accurately stratify people at the extreme ends of the continuum. SRS scores could also be influenced by other factors, such as cognitive level,^46^ although the nature of these relationships is likely much more complex.^47^ This is especially important for our comparisons of twin pairs that were concordant or discordant for having ASD, because they differed in multiple domains in addition to ASD symptom severity, including language and communication skills, IQ, and the presence of other psychiatric symptoms. Second, as with any neuroimaging data sample, participant motion in the scanner may have influenced our scan quality and subsequent data processing and analyses in unknown ways. We do not believe that this is a significant concern for this sample, because all participants were assessed on an MRI simulator prior to data collection, and those who were not within acceptable motion thresholds were provided additional training, offered the use of light procedural sedation with propofol, or excluded from the study. Trained raters also visually inspected all images to confirm sufficient image quality, and statistical analyses were used to identify and exclude extreme outliers. There are also limitations of the MRI sequences that were used and choice of analysis parameters and software.^48^ Third, our novel sample is rather large for an MRI study of ASD, yet also rather small for applying advanced twin modeling approaches, especially for accurately assessing the fit of the classical ACE model. This basic approach also assumes that shared environmental effects are the same for both MZ and DZ twins and that there are no gene-by-environment interactions, which may result in the allocation of some environmental variance to the genetic factor. Using the ACE model without including parent data may also further underestimate some shared environmental influences and contribute to the lack of good model fit. In addition, our new Twin-Pair Difference-score analytical method will require replication and validation in other samples. Finally, the wide age range of this twin sample and cross-sectional comparison of children and adolescents with and without ASD did not support adequate examination of age-related changes in white matter, such that it will be important to account for developmental changes in these relationships and to identify critical periods to further refine this line of investigation.

Given increased awareness of the impact that some environmental factors have in autism,^8,9^ new investigations to identify the critical developmental periods for these complex interactions (eg, prenatal^49^ vs postnatal^50^), the exact nature of these environmental factors,^51–55^ and their differential effects on the brain^10–12^ will be paramount for advancing the development of neurobiological models for ASD. This line of investigation can also be further enhanced by using new analytical methods, including the Twin-Pair Difference-Score and Contribution to Pathway Covariance analyses presented herein. This is especially important for disentangling how complex genetic architecture interacts with specific environmental factors to mediate neurobiological variation across individuals. Potential environmental influences that have been identified to date include a wide range of factors such as parental age,^51^ maternal^52^ or congenital^53^ infection, and toxin^54^ or pollution^55^ exposure, but clearly not vaccination status.^56^ Further research into the role of these and other environmental factors in ASD is particularly promising in regard to potential treatments, because some environmental factors may be more amenable to intervention than other etiological contributions. Alternatively, the effects of having autism on one’s own environment or brain plasticity in response to different treatments may also account for some of the variation attributed to environmental factors in this study.^57,58^ Either way, it is possible that patients with core autism-related symptoms who are seeking treatments to improve mental health and wellness may be able to gain positive long-term clinical outcomes through changes to their environment that modulate the neurobiology underlying important brain–behavior relationships.

## Supplementary Material

MMC1

## Figures and Tables

**TABLE 1:** Demographics and Clinical Characteristics

Demographics, all twin pairs	Autism (n = 50)		Control (n = 34)		Autism vs Control		
	Mean or count	SD	Mean or count	SD	*t* or χ^2^	*p*	FDR
Age, y	10.64	2.55	9.68	2.65	1.67	.10	.20
Sex, male/female	40/10	—	24/10	—	0.99	.32	.40
SES, Hollingshead	51.76	11.00	55.71	8.76	−1.71	.09	.20
Ethnicity and race	—	—	—	—	5.15	.40	.40
Asian, Asian American	3		3				
Black, African American	1		0				
Hispanic, Latinx	4		1				
White	30		26				
Other or Multiracial	9		4				
Unknown	3		0				
Clinical characteristics, all individuals	Autism (n = 82)		Control (n = 68)		Autism vs Control		
	Mean or count	SD	Mean or count	SD	*t* or χ^2^	*p*	FDR
Handedness (right/left)	69/13	—	62/6	—	1.66	.20	.20
Full Scale IQ	82.42	26.30	113.91	11.62	−9.14	<.001*	<.001**
SRS	71.33	16.25	43.69	5.47	13.30	<.001*	<.001**
ADI-R Diagnostic Total	40.18	14.11	—	—	—	—	—
ADOS-2 Comparison Score	7.25	1.79	—	—	—	—	—
Clinical characteristics, only discordant twin pairs	Autism (n = 16)		Co-twins (n = 16)		Autism vs Co-twins		
	Mean or count	SD	Mean or count	SD	*t* or χ^2^	*p*	FDR
Handedness (right/left)	13/3	—	15/1	—	0.25	.63	.63
Full Scale IQ	74.56	32.84	107.25	11.50	−4.34	.001 *	.001 *
SRS	75.00	18.77	46.07	7.36	4.98	<.001*	<.001**
ADI-R Diagnostic Total	45.13	9.09	4.69	6.26	12.31	<.001*	<.001**
ADOS-2 Comparison Score	7.53	1.73	1.40	0.74	14.87	<.001*	<.001**

**Note**: Demographics for all twin pairs (n = pairs) and clinical characteristics for all individuals (n = individuals), excluding twins without autism from discordant pairs, as well as only for twin pairs who were discordant for autism (n = pairs and individuals) are presented. Mean and SD or count within each category are shown. SRS is the total Social Responsiveness Scale score, ADI-R Diagnostic Total is the sum of the Social Interaction, Communication, and Restricted/Repetitive Behavior subscales of the Autism Diagnostic Interview-Revised, and ADOS-2 scores from the Autism Diagnostic Observation Schedule-2 are shown. Group comparisons were conducted with independent samples t tests (t) and **χ**^2^ or paired-samples t tests and **χ**^2^ with the McNemar test. Socioeconomic status (SES) was assessed using the Hollingshead method. Significant difference at

* p < .05 or

** false discovery rate (FDR)^31^ corrected for multiple comparisons.

**TABLE 2:** Comparisons Between Twins With Autism and Control Twins

	Autism (n = 75)		Control (n = 66)		Autism vs control		
White matter tract	Mean	SD	Mean	SD	*t*	*p*	FDR
Fractional anisotropy							
Average							
Commissural fibers	0.504	0.021	0.514	0.015	−4.49	<.001*	.004**
Corpus callosum	0.629	0.032	0.646	0.025	−3.91	<.001*	.004**
Fornix	0.481	0.085	0.462	0.100	0.45	.65	.61
Projection fibers							
Cerebellar peduncles	0.511	0.027	0.522	0.021	−2.60	.01*	.02**
Corona radiata	0.442	0.022	0.448	0.016	−2.47	.02*	.03**
Corticospinal tract	0.536	0.051	0.562	0.036	−2.86	.01*	.01**
Internal capsule	0.578	0.024	0.587	0.018	−3.31	.002*	.004**
Posterior thalamic radiation	0.496	0.043	0.515	0.035	−3.22	.002*	.01**
Association fibers							
Cingulum	0.372	0.048	0.360	0.040	0.51	.61	.61
External capsule	0.380	0.025	0.389	0.022	−2.90	.01*	.01**
Sagittal stratum	0.445	0.038	0.448	0.027	−1.37	.18	.21
Superior fronto–occipital	0.488	0.056	0.493	0.062	−1.04	.30	.35
Superior longitudinal	0.416	0.030	0.420	0.030	−1.73	.09	.12
	Autism (n = 76)		Control (n = 64)		Autism vs Control		
White matter tract	Mean	SD	Mean	SD	*t*	*p*	FDR
Mean diffusivity × 10^−3^							
Average	0.954	0.095	0.943	0.087	1.23	.22	.49
Commissural fibers							
Corpus callosum	1.254	0.349	1.205	0.313	0.95	.35	.57
Fornix	1.454	0.240	1.449	0.244	0.13	.90	.90
Projection fibers							
Cerebellar peduncles	1.111	0.157	1.115	0.210	−0.45	.66	.71
Corona radiata	0.832	0.053	0.818	0.034	2.61	.01*	.16
Corticospinal tract	1.065	0.312	1.005	0.340	1.06	.29	.54
Internal capsule	0.788	0.037	0.791	0.036	0.72	.47	.61
Posterior thalamic radiation	0.940	0.155	0.914	0.099	0.75	.46	.61
Association fibers							
Cingulum	0.824	0.042	0.822	0.041	1.71	.09	.40
External capsule	0.815	0.039	0.818	0.035	1.22	.23	.49
Sagittal stratum	0.904	0.057	0.898	0.052	0.66	.52	.61
Superior fronto-occipital	0.748	0.034	0.744	0.022	1.40	.17	.49
Superior longitudinal	0.786	0.040	0.783	0.034	2.07	.04*	.28

**Note:** All twins with autism were compared to all control twins, and twins without autism from discordant twin pairs were excluded. Group comparisons of average and tract-specific fractional anisotropy and mean diffusivity were completed using linear mixed modeling adjusting for the fixed effects of age, sex, and diagnosis and the random effects of family and zygosity. Significant comparison at

* p < .05 or

** false discovery rate (FDR)^31^ corrected across all comparisons. All of the group-related differences shown above remained significant after adjusting for full-scale IQ.

**TABLE 3:** ACE Models in Twins Concordant for Having Autism and Control Without Twins

	Autism			Control			Autism			Control			Autism		Control	
			
White matter tract	A	95 % CI	*p*	A	95% CI	*p*	C	95% CI	*p*	C	95% CI	*p*	E	95% CI	E	95% CI
Fractional anisotropy																
Average	0.71	[0.33,1.09]	<.001**	0.80	[0.57,1.02]	<.001**	X	X	X	X	X	X	0.29	[−0.09,0.77]	0.20	[−0.02,0.43]
Commissural fibers																
Corpus callosum	—	—	—	0.55	[0.25,0.86]	<.001**	X	X	X	X	X	X	—	—	0.45	[0.14,0.75]
Fornix	0.50	[0.15,0.85]	01**	—	—	—	X	X	X	X	X	X	0.50	[0.15,0.85]	—	—
**Projection fibers**																
Cerebellar peduncles	X	X	X	X	X	X	0.56	[0.28,0.85]	<.001**	0.41	[0.07,0.75]	.02**	0.44	[0.15,0.72]	0.59	[0.25,0.93]
Corona radiata	0.76	[0.42,1.11]	<.001**	0.62	[0.22,1.01]	.002**	X	X	X	X	X	X	0.24	[−0.11,0.58]	0.38	[−0.01,0.78]
Corticospinal tract	X	X	X	X	X	X	0.77	[0.54,1.00]	<.001**	0.52	[0.17,0.87]	.004**	0.23	[0.00,0.46]	0.48	[0.13,0.83]
Internal capsule	X	X	X	0.75	[0.51,0.99]	<.001**	0.65	[0.43,0.87]	<.001**	X	X	X	0.35	[0.01,0.49]	0.25	[0.01,0.49]
Posterior thalamic radiation	X	X	X	0.74	[0.50,0.98]	<.001**	0.55	[0.32,0.79]	<.001**	X	X	X	0.45	[0.02,0.50]	0.26	[0.02,0.50]
**Association fibers**																
Cingulum	—	—	—	0.52	[0.16,0.88]	.004**	X	X	X	X	X	X	-	-	0.48	[0.12,0.84]
External capsule	X	X	X	0.76	[0.47,1.05]	<.001**	0.34	[−0.02,0.69]	.07	X	X	X	0.66	[0.31,1.02]	0.24	[−0.05,0.53]
Sagittal stratum	X	X	X	—	—	—	0.60	[0.27,0.92]	<.001**	X	X	X	0.40	[0.08,0.73]	—	-
Superior fronto- occipital	0.21	[−0.23,0.65]	.36	—	—	—	X	X	X	X	X	X	0.79	[0.35,1.23]	—	—
Superior longitudinal	X	X	X	X	X	X	0.67	[0.43,0.92]	<.001**	0.74	[0.59,0.88]	<.001**	0.33	[0.08,0.57]	0.26	[0.12,0.41]
**Mean diffusivity**																
Average	0.84	[0.32,1.36]	.002**	0.80	[0.55,1.04]	<.001**	X	X	X	X	X	X	0.16	[−0.36,0.68]	0.20	[−0.04,0.45]
Commissural fibers																
Corpus callosum	X	X	X	X	X	X	0.64	[0.26,1.01]	.001**	0.48	[0.14,0.81]	01**	0.36	[−0.01,0.74]	0.52	[0.19,0.86]
Fornix	0.49	[0.08,0.90]	.02**	X	X	X	X	X	X	0.48	[0.21,0.76]	.001**	0.51	[0.10,0.92]	0.52	[0.24,0.79]
Projection fibers																
Cerebellar peduncles	0.54	[0.06,1.02]	.03**	X	X	X	X	X	X	0.76	[0.52,1.00]	<.001**	0.46	[−0.02,0.94]	0.24	[0.00,0.48]
Corona radiata	0.94	[0.54,1.35]	<.001**	0.73	[0.44,1.02]	<.001**	X	X	X	X	X	X	0.06	[−0.35,0.46]	0.27	[−0.02,0.56]
Corticospinal tract	X	X	X	X	X	X	0.70	[0.43,0.96]	<.001**	0.59	[0.18,1.01]	01**	0.30	[0.04,0.57]	0.41	[−0.01,0.82]
Internal capsule	0.49	[0.04,0.94]	.03**	0.82	[0.64,0.99]	<.001**	X	X	X	X	X	X	0.51	[0.06,0.96]	0.18	[0.01,0.46]
Posterior thalamic radiation	X	X	X	X	X	X	0.66	[0.39,0.94]	<.001**	0.70	[0.21,1.19]	01**	0.34	[0.06,0.61]	0.30	[−0.19,0.79]
Association fibers																
Cingulum	X	X	X	0.76	[0.52,1.00]	<.001**	0.71	[0.53,0.88]	<.001**	X	X	X	0.29	[0.12,0.47]	0.24	[0.00,0.48]
External capsule	0.70	[0.34,1.06]	<.001**	X	X	X	X	X	X	0.70	[0.53,0.88]	<.001**	0.30	[−0.06,0.66]	0.30	[0.12,0.47]
Sagittal stratum	0.54	[0.09,0.99]	.02**	0.57	[0.20,0.94]	.002**	X	X	X	X	X	X	0.46	[0.01,0.91]	0.43	[0.06,0.80]
Superior fronto-occipital	X	X	X	X	X	X	0.78	[0.65,0.90]	<.001**	0.39	[0.09,0.69]	.01**	0.22	[0.10,0.35]	0.61	[0.31,0.91]
Superior longitudinal	0.97	[0.59,1.35]	<.001**	X	X	X	X	X	X	0.74	[0.58,0.90]	<.001**	0.03	[−0.35,0.41]	0.26	[0.10,0.42]

**Note:** The ACE model for broad sense heritability, additive genetic (A) and common/shared (C) or unique/unshared (E) environmental factors, was calculated separately for all twin pairs in which both twins were diagnosed with autism spectrum disorder or both were control twins without psychiatric or neurological diagnoses. Twin pairs discordant for autism spectrum disorder were excluded. When A or C was non-significant within the model (X), a constrained AE or CE model was used instead. When model assumptions were violated, model estimates were not generated, but the parameters that would have been most appropriate are indicated (—). E estimates were based on the residuals and were not tested for significance. Significant model parameter at

* p < .05 or

** false discovery rate (FDR)^31^ correction across all of the estimates within each column for each measure are indicated.
