# Supplementary material for: A Twin Study of Altered White Matter Heritability in Youth With Autism Spectrum Disorder
Source: J Am Acad Child Adolesc Psychiatry. Author manuscript; Available in PMC 2024 Feb 16. (PMC10802971; doi:10.1016/j.jaac.2023.05.030)
Supplement: MMC1 [file NIHMS1927481-supplement-MMC1.pdf]

SUPPLEMENT 1

Supplemental Methods and Materials

Cognitive and Behavioral Testing

Clinical diagnoses of autism spectrum disorder (ASD) were confirmed using the Autism Diagnostic Observation Schedule, 2nd Edition,[1] a direct observation of the child's behaviors, and the Autism Diagnostic Interview-Revised,[2] a structured parent interview regarding developmental history. These measures are considered gold-standard for research confirmation of clinical or community diagnoses of ASD. The Autism Diagnostic Observation Schedule is composed of four separate modules, one of which is administered depending on the participants age and language level. Module One was used for the 17% of our ASD sample who had limited speech, Module Two was used for the 6% who had some speech but were not verbally fluent, Module Three was used for the 74% of children who were verbally fluent, and Module Four was used for the 3% of adolescents who were verbally fluent. Thus, our sample was comprised of autistic twins who exhibited a wide range of cognitive and behavioral skills.

The Social Responsiveness Scale (SRS),[3] a parent report checklist of autism-related symptoms, was also obtained to compare core symptom severity between twin pairs. The SRS was designed to provide a continuous measure of social ability (from impaired to above average) and autistic mannerisms. Higher scores are associated with more severe autism-related symptoms. The updated social communication impairment and restricted, repetitive behavior factor structure[4] was used to compare symptom severity between groups and examine important brain-behavior relationships. The Stanford-Binet Intelligence Scales, 5th Edition[5] was used to assess general cognitive abilities in 98% of the participants both with and without ASD and provided subscale scores for full-scale, verbal, and non-verbal intelligence quotients (IQ). The remaining participants completed the Mullen Scales of Early learning to assess non-verbal cognitive abilities because it was more appropriate for their language and communication skills.[6,7]

SRS scores have been associated with language and communication skills as well as other factors such as age, general cognitive abilities and IQ, and non-autism related psychiatric symptoms.[8] However, the nature of these relationships is likely much more complex,[9] especially considering the potential genetic overlap of autism symptoms with other psychiatric domains[10] and cognitive abilities.[11] Our basic assessment of brain-behavior correlations separately for symptom severity and IQ should help indicate whether these relationships are similar or different across domains in the current sample. Overall, we believe that the range of cognitive, language, and adaptive skills in our neuroimaging twin sample is a strength of this study that increases the impact of our findings.

Magnetic Resonance Imaging

Neuroimaging was conducted on General Electric 3T MR750 scanners (Waukesha, Wisconsin, USA) using standard 8-channel head coils. All participants were assessed on a magnetic resonance imaging (MRI) simulator prior to data collection and any participants that were not within acceptable motion thresholds (< 2mm in any direction) were provided additional training, offered the use of light procedural sedation, or excluded from the study. Autistic participants that were unable to remain motionless were administered propofol under the supervision of an anesthesiologist at a rate of 200-300 mcg/kg/min. Four separate spin-echo, echo-planar diffusion-weighted product sequences were acquired with the following parameters: field of view= 24 cm, matrix size= 128x128, echo time= minimum, response time= 5700 msec, 45 axial slices, slice thickness= 2.9 mm, skip= 0. Twenty-five diffusion directions were acquired with diffusion weighting b= 1000 sec/mm^2. Diffusion weighted imaging (DWI) is a MRI approach that calculates the rate of tissue water diffusion per voxel.[12] Because the rate of tissue water diffusion is highly determined by the architecture of the tissue, it is possible to infer differences in tissue architecture (e.g., integrity, dimensions) by modeling diffusion tensor imaging (DTI) rates. There are several diffusion rates that can be calculated per voxel, of which fractional anisotropy (FA) and mean diffusivity (MD) are the most common. FA at each voxel is the relative difference between the longest diffusion eigenvector as compared to the lengths of the other eigenvectors of diffusion within the voxel, thereby demonstrating the directional preference of diffusion. An FA value of 1 would suggest linear (anisotropic) diffusion. MD at each voxel is simply the average of length of the diffusion eigenvectors within each voxel.

Trained raters visually inspected all images before inclusion and then data within each participant were co-registered to the first good quality repetition. Processing procedures were conducted using NiftyReg [13] and the FMRIB Software Library Diffusion Toolbox using Tract-Based Spatial Statistics.[14,15] Specifically, non-diffusion weighted (b0) images from different repetitions were linearly co-registered to the b0 image from the first repetition using "reg_aladin" function from the NiftiReg software http://cmictig.cs.ucl.ac.uk/wiki/index.php/NiftyReg.[13] To compensate for the minor residual non-linear deformation between b0 images, b0 images from different repetitions were non-linearly co-registered to the b0 image from the first repetition using NiftiReg's "reg_f3d" function, with the obtained affine transformation as the initialization. The resultant non-linear transformations were applied to the diffusion data (b0 and DWIs) of each corresponding repetition. The diffusion encoding directions of DWIs were rotated using the "fdt_rotate_bvecs" function from the FMRIB Software Library (FSL, http://fsl.fmrib.ox.ac.uk/fsl/fslwiki/) [16] based on the obtained affine transformations. The co-registered repetitions of diffusion MRI data were then combined. The combined diffusion data were corrected for eddy current distortions and bulk motion using FSL's "eddy_cuda" function with 32 iterations. Diffusion tensor model fitting was performed using FSL's "dtifit" to derive the FA map and the voxel-wised primary diffusion orientations. The primary measures of interest were FA and MD.

FA maps were visually-inspected for artifacts and data processing errors and then registered to the most representative target image from our pediatric dataset using Tract-Based Spatial Statistics.[14] Subject-specific transformations were then applied to the MD maps and all images were registered to standard space, skeletonized, and segmented into independent tracts from the John Hopkins University white matter parcellation atlas,[17] as outlined in the ENIGMA protocol reported in Jahanshad et al. 2013,[18] see Figure 1 in the main text. We chose this approach in order to compare our data to heritability estimates from previous twin studies while also minimizing the number of regions of interest for multiple comparisons. To further reduce the number of comparisons, we also combined the genu, body, and splenium of the corpus callosum into a single region and did not examine the inferior longitudinal fasciculus or inferior fronto-occipital fasciculus separately because they were combined into the sagittal stratum. Based on our preliminary findings,[19] we also added the cerebellar peduncles. Our final regions of interest were grouped into three categories: 1) commissural fibers –connect the two hemispheres of brain and include the corpus callosum and fornix; 2) projection fibers – connect the cortex

with other parts of the brain and include the cerebellar peduncles, corona radiata, corticospinal tract, internal capsule, and posterior thalamic radiation; and 3) association fibers – connect areas of the cortex within the same hemisphere and include the cingulum, external capsule, sagittal stratum, superior fronto-occipital fasciculus, and superior longitudinal fasciculus. Tract-based FA and MD were then calculated as averages across all voxels within each tract and were examined for extreme statistical outliers using Tukey's method.[20]

Statistical Analyses

Twin modeling was carried out using two distinct types of analyses for this study.

Classical ACE twin model analyses. The first type of twin analysis fit the classical ACE variance-component twin model, which traditionally estimates the Proportion of Trait Variance (PTV) attributable to each of three sources: (A) additive genetic effects; (C) common environmental effects preferentially shared by both members of a twin pair; and (E) unique environmental effects unshared by co-twins. To fit the classical ACE twin model, we used Generalized DeFries-Fulker (DF) Regression, described in detail elsewhere,[21,22] and implemented it using STATA®. Generalized DF regression uses an iterative regression model applied to monozygotic (MZ) and dizygotic (DZ) twin data to fit the ACE model in an unbiased way. Bootstrap analysis with 1,000 replications was used to compute statistical significance and confidence intervals. When A or C was initially non-significant in the model, we applied sequential elimination and fit the more parsimonious AE or CE model.

Twin-pair difference-score analyses. The second type of twin analysis explores the putative genetic or environmental pathway underlying a pair of shared traits or phenotypes, which in this case are comprised of one brain measure (e.g., FA in the corpus callosum) and one behavioral measure (e.g., social communication impairments on the SRS). The novel method used here utilizes twin-pair difference-scores (TPDSs) to extend and modify the "MZ difference score" method first proposed by Pike,[23] which was an application to MZ twins of the classic statistical matched-pair design that has since been used by a number of authors, including ourselves.[24-28] To our knowledge, we present the first full mathematical rationale for the use of difference scores in the twin model context. The resulting, expanded TPDS method used in our paper adds to the original MZ difference score method in three ways: 1) it incorporates information from DZ twins, 2) it relies on covariances instead of correlations, and as a consequence, 3) it provides precise quantitative estimates of the Contribution to Pathway Covariance (CPC) from both additive genetic, A, or unique or unshared environmental factors, E. Thus, TPDS analysis allows for a comparative evaluation of the role of genetic and unshared environmental pathways within and between different pairs of traits.

The TPDS rationale. Prior to conducting a TPDS analysis, we standardize all trait variables (subtracting the mean and dividing by the standard deviation of each variable) to ensure comparability of scale. For random trait variables X and Y, we calculate twin-pair differences scores for each twin pair as $X_1 – X_2$ and $Y_1 – Y_2$, respectively, where X_i is the value of X for twin the ith twin and Y_i is the value of Y for i^{th} twin. Either twin in a pair can be designated as twin 1 or 2, but the same order should be used throughout. Using standard covariance calculations, the covariance of the difference scores of X and Y can be written as:

$$\text{Cov}(X_1 – X_2, Y_1 – Y_2) = \text{Cov}(X_1, Y_1) – \text{Cov}(X_1, Y_2) – \text{Cov}(X_2, Y_1) + \text{Cov}(X_2, Y_2)$$

$$= 2\,\text{Cov}(X_1, Y_1) – 2\,\text{Cov}(X_1, Y_2).$$

The derivation of the TPDS method depends on a decomposition similar to that used to derive the classical univariate ACE model above. Let the expected value of X in a given individual be written as $b_1A + b_2 C + b_3 E + b_4 U$. Herein, A, C, and E encompass all shared latent factors or variables in the pathway connecting traits X and Y. Specifically, A represents genetic variants acting in an additive way; C represents environmental variables shared in common by co-twins; and E represents environmental variables not shared by co-twins. The final term U represents any variable that affects X but not Y, including a random error term. The b's are regression coefficients relating X to A, C, E and U, respectively. An analogous decomposition of Y as $d_1A + d_2 C + d_3 E + d_4 W$, uses distinct regression coefficients labelled by d's. Note that U and W drop from the following argument as they do not contribute to the X-Y covariance by definition. Let V(A), V(C) and V(E) be the variances of A, C and E, respectively. It follows that, independent of zygosity, the total within-twin covariance of X and Y is

$$Cov\ (X_1,Y_1) = b_1 d_1 V(\ A\ ) + b_2 d_2 V(\ C\ ) + b_3 d_3 V(\ E\ )$$

The three constituent terms above are the CPCs from genetic, shared environmental and non-shared environmental sources, respectively.

Conversely, cross-twin trait covariances do depend on zygosity. Specifically for MZ pairs,

$$Cov\ (X_1, Y_2) = b_1 d_1 V(\ A\ ) + b_2 d_2 V(\ C\ )$$

as MZ co-twins share 100% of both their genetics and shared environment. For DZ pairs,

$$Cov\ (X_1, Y_2) = b_1 d_1 V(\ A\ ) / 2 + b_2 d_2 V(\ C\ )$$

as DZ co-twins share 50% of their genetics on average. We can now obtain covariances for the difference scores. For MZ twin pairs, this covariance is

$$Cov_{MZ}\ (X_1\text{-}X_2, Y_1\text{-}Y_2)$$

$$= 2\ [\ b_1 d_1 V(\ A\ ) + b_2 d_2 V(\ C\ ) + b_3 d_3 V(\ E\ )\ ] – 2\ [\ b_1 d_1 V(\ A\ ) + b_2 d_2 V(\ C\ )\ ].$$

$$= 2\ b_3 d_3 V(\ E\ ).$$

and the DZ difference-score covariance is

$$Cov_{DZ}\ (X_1\text{-}X_2, Y_1\text{-}Y_2\ )$$

$$= 2\ [\ b_1 d_1 V(\ A\ ) + b_2 d_2 V(\ C\ ) + b_3 d_3 V(\ E\ )\ ]$$

$$–\ 2\ [\ b_1 d_1\ V(\ A\ ) / 2 + b_2 d_2 V(\ E\ )\ ]$$

$$= b_1 d_1\ V(A) + 2\ b_3 d_3 V(\ E\ ).$$

Thus, twin-pair difference-scores can be used to estimate the non-shared environmental CPC between traits X and Y as

$$b_3 d_3 V(\ E\ ) = Cov_{MZ}\ (X_1\text{-}X_2, Y_1\text{-}Y_2\ ) / 2$$

and the additive genetic CPC between the same traits as

$$b_1 d_1 V(A) = Cov_{DZ} (X_1\text{-}X_2, Y_1\text{-}Y_2\ ) - Cov_{MZ}\ (\ X_1\text{-}X_2, Y_1\text{-}Y_2\ )$$

The estimates may be positive or negative and may have opposite signs, suggesting that the two CPCs act to offset each other. Statistical significance and/or confidence intervals can be obtained using the bootstrap or other statistical methods. A significant non-zero covariance between the MZ difference-scores of two traits is interpreted to mean that both traits are associated with at least some of the same latent environmental factors. Significant inequality of MZ and DZ difference-score covariances can be interpreted to mean that both traits are associated with at least some of the same unobserved genetic factors. However, one cannot infer causal direction from the model alone.

Supplemental Results

Table S1. Group comparisons within twin pairs discordant for autism.

White Matter Tract	***Autism*** *(n=15)*		***Co-twins*** *(n=15)*				
	M	SD	M	SD	t	p	FDR
**Fractional Anisotropy**							
Average	0.495	0.02	0.507	0.03	-2.54	0.02*	0.12
Commissural Fibers							
Corpus Callosum	0.618	0.03	0.641	0.03	-2.92	0.01*	0.12
Fornix	0.429	0.08	0.466	0.11	-1.23	0.24	0.42
Projection Fibers							
Cerebellar Peduncles	0.510	0.03	0.508	0.03	0.21	0.83	0.83
Corona Radiata	0.435	0.02	0.446	0.03	-2.46	0.03*	0.12
Corticospinal Tract	0.536	0.06	0.552	0.04	-1.09	0.30	0.43
Internal Capsule	0.570	0.02	0.577	0.03	-1.94	0.07	0.19
Posterior Thalamic Radiation	0.486	0.04	0.501	0.06	-1.18	0.26	0.42
Association Fibers							
Cingulum	0.361	0.04	0.357	0.04	0.35	0.73	0.83
External Capsule	0.366	0.02	0.379	0.03	-1.96	0.07	0.19
Sagittal Stratum	0.434	0.03	0.442	0.03	-0.95	0.36	0.46
Superior Fronto-occipital Fasciculus	0.486	0.06	0.482	0.05	1.61	0.13	0.28
Superior Longitudinal Fasciculus	0.408	0.02	0.409	0.04	-0.25	0.81	0.83
	Autism *(n=16)*		***Co-twins*** *(n=16)*				
**Mean Diffusivity x 10^{-3}**							
Average	1.000	0.11	0.940	0.08	2.15	0.05*	0.12
Commissural Fibers							
Corpus Callosum	1.420	0.42	1.220	0.21	2.11	0.05	0.12
Fornix	1.520	0.16	1.450	0.34	0.95	0.36	0.44
Projection Fibers							
Cerebellar Peduncles	1.120	0.17	1.120	0.17	-0.12	0.91	0.97
Corona Radiata	0.860	0.06	0.820	0.04	2.22	0.04*	0.12
Corticospinal Tract	1.020	0.32	1.030	0.28	-0.04	0.97	0.97
Internal Capsule	0.790	0.03	0.780	0.04	1.93	0.07	0.14
Posterior Thalamic Radiation	0.990	0.27	0.920	0.11	1.00	0.33	0.44
Association Fibers							
Cingulum	0.840	0.02	0.810	0.05	2.28	0.04*	0.12
External Capsule	0.830	0.05	0.810	0.05	2.39	0.03*	0.12
Sagittal Stratum	0.900	0.06	0.880	0.07	0.92	0.37	0.44
Superior Fronto-occipital Fasciculus	0.750	0.02	0.740	0.03	1.62	0.13	0.21
Superior Longitudinal Fasciculus	0.790	0.03	0.780	0.04	2.08	0.06	0.12

Group comparisons of average and tract-specific fractional anisotropy and mean diffusivity within twin pairs discordant for ASD (n = # of individuals and pairs) were completed using repeated-measures structured covariance models using SAS®.[29] Discordant twin pairs were defined as those in which one twin had a confirmed diagnosis of ASD and the other twin did not exhibit any clinical or sub-threshold symptoms. Significant comparison at * $p < 0.05$ or ** False Discovery Rate [30] corrected across all comparisons. These group-related differences were no longer significant when adjusting for differences in full-scale IQ.

Table S2. Intraclass correlation comparisons between all monozygotic and dizygotic twin pairs, including both concordant and discordant twin pairs.

	All Twin Pairs				***MZ vs DZ***		
White Matter Tract	MZ (n=78)	p	DZ (n=80)	p	z	p	FDR
**Fractional Anisotropy**							
Average	0.74 [0.58,0.90]	<0.001	0.50 [0.23,0.77]	<0.001	2.47	0.01*****	0.03**
Commissural Fibers							
Corpus Callosum	0.57 [0.29,0.85]	<0.001	0.20 [-0.11,0.50]	0.20	2.74	0.01*****	0.02**
Fornix	0.53 [0.23,0.84]	0.001	0.11 [-0.19,0.41]	0.46	2.96	0.003*****	0.02**
Projection Fibers							
Cerebellar Peduncles	0.50 [0.19,0.80]	0.001	0.58 [0.31,0.84]	<0.001	-0.70	0.48	0.52
Corona Radiata	0.72 [0.52,0.92]	<0.001	0.49 [0.21,0.76]	<0.001	2.29	0.02*****	0.04**
Corticospinal Tract	0.64 [0.36,0.92]	<0.001	0.55 [0.32,0.77]	<0.001	0.86	0.39	0.46
Internal Capsule	0.74 [0.59,0.89]	<0.001	0.61 [0.43,0.80]	<0.001	1.49	0.14	0.22
Posterior Thalamic Radiation	0.55 [0.34,0.77]	<0.001	0.54 [0.32,0.77]	<0.001	0.09	0.93	0.93
Association Fibers							
Cingulum	0.60 [-0.15,0.49]	<0.001	0.17 [-0.15,0.49]	0.31	3.21	0.001*****	0.01**
External Capsule	0.66 [0.41,0.91]	<0.001	0.37 [0.11,0.62]	0.01	2.49	0.01*****	0.03**
Sagittal Stratum	0.52 [0.29,0.75]	<0.001	0.40 [0.07,0.74]	0.02	0.94	0.35	0.45
Superior Fronto-occipital	0.39 [0.15,0.64]	0.002	-0.04 [-0.24,0.17]	0.73	2.78	0.01*****	0.02**
Superior Longitudinal	0.73 [0.56,0.90]	<0.001	0.64 [0.47,0.80]	<0.001	1.05	0.29	0.43
	MZ (n=72)		DZ (n=86)				
**Mean Diffusivity**							
Average	0.78 [0.62,0.93]	<0.001	0.38 [0.16,0.60]	0.001	3.96	<0.001*****	<0.001**
Commissural Fibers							
Corpus Callosum	0.73 [0.57,0.89]	<0.001	0.43 [0.18,0.69]	0.001	2.88	0.004*****	0.01**
Fornix	0.51 [0.22,0.80]	<0.001	0.34 [0.13,0.54]	0.001	1.28	0.20	0.24
Projection Fibers							
Cerebellar Peduncles	0.77 [0.54,1.00]	<0.001	0.44 [0.17,0.71]	0.002	3.36	<0.001*****	<0.001**
Corona Radiata	0.83 [0.69,0.97]	<0.001	0.26 [-0.01,0.52]	0.054	5.66	<0.001*****	<0.001**
Corticospinal Tract	0.51 [0.19,0.83]	0.002	0.60 [0.30,0.90]	<0.001	-0.80	0.42	0.46
Internal Capsule	0.78 [0.65,0.90]	<0.001	0.29 [-0.003,0.59]	0.052	4.58	<0.001*****	<0.001**
Posterior Thalamic Radiation	0.59 [0.34,0.84]	<0.001	0.28 [-0.10,0.67]	0.148	2.39	0.02*****	0.02**
Association Fibers							
Cingulum	0.77 [0.62,0.92]	<0.001	0.42 [0.14,0.71]	0.004	3.51	<0.001*****	0.001**
External Capsule	0.76 [0.63,0.89]	<0.002	0.54 [0.20,0.88]	0.002	2.41	0.02*****	0.02**
Sagittal Stratum	0.58 [0.31,0.85]	<0.001	0.27 [-0.02,0.56]	0.063	2.37	0.02*****	0.02**
Superior Fronto-occipital	0.66 [0.52,0.80]	<0.001	0.63 [0.43,0.84]	<0.001	0.32	0.75	0.75
Superior Longitudinal	0.81 [0.68,0.94]	<0.001	0.54 [0.29,0.79]	<0.001	3.21	0.001*****	0.002**

Intra-class correlation coefficients are compared with Fishers r to z transformation between monozygotic (MZ) and dizygotic (DZ) twin pairs (n = # of pairs) for all participants, adjusted for diagnosis and sex. Significant group comparison at ***** p < 0.05 or ** False Discovery Rate[30] correction across the tests within each column for each measure.

Table S3. Intraclass correlation comparisons between twin pairs concordant for having autism and controls without autism.

	Autism				***Control***				***Autism vs Control***					
Structure	MZ (n=30)	p	DZ (n=28)	p	MZ (n=40)	p	DZ (n=26)	p	z(MZ)	p	FDR	z(DZ)	p	FDR
**Fractional Anisotropy**														
Average	0.65 [0.37,0.94]	<0.001	0.43 [-0.08,0.95]	0.10	0.80 [0.61,0.99]	<0.001	0.40 [-0.03,0.82]	0.07	-1.28	0.20	0.52	0.13	0.90	0.09
Commissural Fibers														
Corpus Callosum	0.46 [-0.08,1.00]	0.09	-0.04 [-0.53,0.45]	0.86	0.60 [0.29,0.91]	<0.001	0.15 [-0.37,0.66]	0.57	-0.77	0.44	0.78	-0.66	0.51	0.62
Fornix	0.58 [0.25,0.91]	0.001	0.16 [-0.29,0.61]	0.50	0.54 [0.07,1.01]	0.03	-0.13 [-0.72,0.46]	0.66	0.23	0.82	0.87	1.01	0.31	0.62
Projection Fibers														
Cerebellar Peduncles	0.53 [0.04,1.02]	0.03	0.61 [0.32,0.91]	<0.001	0.46 [-0.02,0.94]	0.06	0.33 [-0.14,0.81]	0.17	0.37	0.71	0.84	1.27	0.20	0.62
Corona Radiata	0.72 [0.44,1.01]	<0.001	0.44 [-0.01,0.94]	0.08	0.63 [0.23,1.02]	0.002	0.29 [-0.21,0.80]	0.26	0.66	0.51	0.78	0.60	0.55	0.62
Corticospinal Tract	0.83 [0.49,1.16]	<0.001	0.68 [0.34,1.02]	<0.001	0.49 [-0.02,0.99]	0.06	0.58 [0.14,1.01]	0.01	2.58	0.01*****	0.07	0.58	0.56	0.62
Internal Capsule	0.65 [0.34,0.97]	<0.001	0.65 [0.28,1.01]	0.001	0.73 [0.49,0.97]	<0.001	0.41 [0.06,0.77]	0.02	-0.61	0.54	0.78	1.18	0.24	0.62
Posterior Thalamic Radiation	0.40 [-0.01,0.81]	0.06	0.68 [0.39,0.97]	<0.001	0.73 [0.53,0.93]	<0.001	0.39 [-0.15,0.92]	0.16	-2.00	0.05*****	0.16	1.44	0.15	0.62
Association Fibers														
Cingulum	0.83 [0.68,0.97]	<0.001	-0.07 [-0.58,0.45]	0.79	0.54 [0.14,0.93]	0.01	0.16 [-0.35,0.67]	0.53	2.31	0.02*****	0.09	-0.80	0.42	0.62
External Capsule	0.32 [-0.17,0.80]	0.20	0.35 [-0.13,0.83]	0.15	0.82 [0.56,1.08]	<0.001	0.14 [-0.26,0.53]	0.51	-3.26	0.001*****	0.01**	0.78	0.44	0.62
Sagittal Stratum	0.51 [0.11,0.91]	0.01	0.63 [0.20,1.07]	0.004	0.51 [0.18,0.85]	0.003	-0.04 [-0.56,0.47]	0.87	0.00	1.00	1.00	2.70	0.01*****	0.62
Superior Fronto-occipital	0.27 [-0.17,0.71]	0.23	0.02 [-0.52,0.57]	0.93	0.51 [0.20,0.83]	0.002	0.18 [-0.48,0.13]	0.26	-1.13	0.26	0.56	-0.56	0.58	0.62
Superior Longitudinal	0.76 [0.46,1.05]	<0.001	0.63 [0.25,1.01]	0.001	0.71 [0.48,0.95]	<0.001	0.76 [0.55,0.97]	<0.001	0.43	0.67	0.84	-0.88	0.38	0.90
	MZ (n=26)		DZ (n=32)		MZ (n=38)		DZ (n=26)							
**Mean Diffusivity**														
Average	0.76 [0.44,1.07]	<0.001	0.46 [0.004,0.92]	0.048	0.70 [0.45,0.95]	<0.001	0.49 [0.21,0.77]	0.001	0.48	0.63	0.75	-0.14	0.89	0.89
Commissural Fibers														
Corpus Callosum	0.76 [0.45,1.08]	<0.001	0.61 [0.17,1.04]	0.006	0.59 [0.30,0.87]	<0.001	0.43 [-0.04,0.90]	0.070	1.19	0.23	0.75	0.89	0.37	0.61
Fornix	0.58 [0.12,1.05]	0.014	0.13 [-0.25,0.52]	0.501	0.46 [0.08,0.84]	0.019	0.57 [0.19,0.95]	0.003	0.62	0.54	0.75	-1.85	0.06	0.21
Projection Fibers														
Cerebellar Peduncles	0.76 [0.41,1.12]	<0.001	0.001 [-0.56,0.56]	0.996	0.76 [0.44,1.07]	<0.001	0.76 [0.40,1.11]	<0.001	0.00	1.00	1.00	-3.56	<0.001*****	0.01**
Corona Radiata	0.83 [0.55,1.10]	<0.001	0.53 [0.15,0.91]	0.006	0.74 [0.43,1.05]	<0.001	0.35 [-0.01,0.71]	0.056	0.89	0.37	0.75	0.80	0.42	0.61
Corticospinal Tract	0.66 [0.35,0.97]	<0.001	0.71 [0.28,1.15]	0.001	0.45 [-0.08,0.98]	0.095	0.69 [0.22,1.17]	0.004	1.15	0.25	0.75	0.14	0.89	0.89
Internal Capsule	0.70 [0.35,1.05]	<0.001	0.08 [-0.38,0.54]	0.727	0.78 [0.62,0.93]	<0.001	0.51 [0.16,0.86]	0.005	-0.66	0.51	0.75	-1.73	0.08	0.22
Posterior Thalamic Radiation	0.66 [0.34,0.99]	<0.001	0.67 [0.28,1.05]	0.001	0.56 [0.15,0.97]	0.008	0.75 [-0.11,1.61]	0.086	0.60	0.55	0.75	-0.58	0.56	0.73
Association Fibers														
Cingulum	0.73 [0.39,1.08]	<0.001	0.70 [).49,0.90]	<0.001	0.81 [0.66,0.95]	<0.001	0.32 [-0.13,0.78]	0.166	-0.74	0.46	0.75	1.92	0.06	0.21
External Capsule	0.75 [0.50,1.00]	<0.001	0.30 [-0.16,0.77]	0.206	0.79 [0.59,0.98]	<0.001	0.60 [0.31,0.90]	<0.001	-0.37	0.71	0.77	-1.37	0.17	0.37
Sagittal Stratum	0.49 [0.04,0.95]	0.033	0.31 [-0.14,0.75]	0.179	0.59 [0.22,0.97]	0.002	0.21 [-0.38,0.80]	0.485	-0.53	0.60	0.75	0.38	0.70	0.83
Superior Fronto-occipital	0.70 [0.50,0.90]	<0.001	0.83 [0.68,0.99]	<0.001	0.47 [0.18,0.76]	0.001	0.36 [-0.08,0.80]	0.105	1.33	0.18	0.75	2.91	0.004*****	0.03**
Superior Longitudinal	0.86 [0.68,1.04]	<0.001	0.56 [0.16,0.97]	0.007	0.77 [0.56,0.98]	<0.001	0.72 [0.47,0.97]	<0.001	1.02	0.31	0.75	-0.98	0.33	0.61

Intra-class correlation coefficients, adjusted for sex, are compared with Fishers r to z transformation between monozygotic (MZ) and dizygotic (DZ) twin pairs (n = # of pairs) in which both twins were diagnosed with autism spectrum disorder (ASD) or were controls without any neuropsychiatric diagnoses. Twin pairs discordant for ASD were excluded. Significant group comparison at ***** p < 0.05 or ** False Discovery Rate[30] corrected across the tests within each column for each measure are indicated.

Table S4. Contribution to pathway covariance for additive genetic effects on the shared traits between general cognitive abilities and fractional anisotropy.

	All TWIN PAIRS				CONTROL PAIRS				ASD PAIRS				CONCORDANT ASD PAIRS			
**Full Scale IQ**	Obs. Coef.	P>|z|	95% Conf. Interval		Obs. Coef.	P>|z|	95% Conf. Interval		Obs. Coef.	P>|z|	95% Conf. Interval		Obs. Coef.	P>|z|	95% Conf. Interval	
Average	0.45	0.001*	0.18	0.70	0.12	0.068	-0.01	0.26	0.52	0.001*	0.19	0.82	0.10	0.419	-0.17	0.34
Commissural Fibers																
Corpus Callosum	0.48	0.001*	0.18	0.77	0.08	0.278	-0.07	0.23	0.65	0.001*	0.25	1.02	0.28	0.076	-0.06	0.56
Fornix	0.30	0.063	0.00	0.64	0.22	0.051	-0.01	0.44	0.45	0.026*	0.05	0.84	-0.06	0.676	-0.32	0.21
Projection Fibers																
Cerebellar Peduncles	0.12	0.419	-0.17	0.40	0.22	0.003*	0.06	0.35	0.07	0.730	-0.31	0.46	-0.13	0.437	-0.45	0.22
Corona Radiata	0.58	<0.001*	0.26	0.88	0.18	0.068	-0.03	0.36	0.63	<0.001*	0.27	0.96	0.19	0.171	-0.11	0.44
Corticospinal Tract	0.11	0.315	-0.10	0.31	-0.02	0.743	-0.12	0.09	0.15	0.334	-0.17	0.46	-0.13	0.401	-0.42	0.20
Internal Capsule	0.23	0.009*	0.06	0.41	0.08	0.163	-0.04	0.20	0.22	0.056	-0.01	0.44	0.01	0.927	-0.22	0.23
Pos. Thalamic Radiation	0.19	0.259	-0.09	0.56	0.01	0.836	-0.12	0.13	0.21	0.350	-0.20	0.69	0.07	0.645	-0.23	0.37
Association Fibers																
Cingulum	-0.14	0.499	-0.54	0.27	0.10	0.492	-0.15	0.40	-0.16	0.604	-0.74	0.46	-0.46	0.189	-1.16	0.15
External Capsule	0.21	0.136	-0.06	0.48	0.03	0.713	-0.13	0.18	0.23	0.277	-0.18	0.64	-0.12	0.594	-0.58	0.31
Sagittal Stratum	0.43	0.014*	0.08	0.77	0.15	0.275	-0.13	0.41	0.43	0.055	-0.01	0.86	0.07	0.726	-0.38	0.46
Superior Fronto-occipital	0.10	0.524	-0.21	0.41	0.10	0.450	-0.16	0.34	-0.07	0.744	-0.49	0.34	-0.16	0.379	-0.54	0.19
Superior Longitudinal	0.28	0.045*	0.00	0.54	0.09	0.346	-0.09	0.28	0.32	0.085	-0.07	0.66	0.08	0.504	-0.17	0.33
**Verbal IQ**																
Average	0.41	0.004*	0.13	0.68	0.01	0.825	-0.09	0.13	0.51	0.003*	0.16	0.84	0.12	0.391	-0.17	0.36
Commissural Fibers																
Corpus Callosum	0.45	0.005*	0.14	0.77	-0.02	0.825	-0.17	0.14	0.65	0.003*	0.22	1.06	0.26	0.093	-0.05	0.54
Fornix	0.16	0.278	-0.11	0.46	-0.15	0.285	-0.44	0.10	0.42	0.030*	0.04	0.80	-0.05	0.733	-0.34	0.23
Projection Fibers																
Cerebellar Peduncles	0.03	0.847	-0.26	0.32	0.10	0.188	-0.05	0.24	0.01	0.959	-0.38	0.44	-0.11	0.561	-0.45	0.31
Corona Radiata	0.54	<0.001*	0.24	0.84	0.13	0.123	-0.04	0.29	0.60	0.001*	0.24	0.91	0.17	0.247	-0.15	0.42
Corticospinal Tract	0.13	0.221	-0.08	0.34	-0.08	0.235	-0.21	0.04	0.25	0.098	-0.04	0.55	-0.07	0.615	-0.35	0.23
Internal Capsule	0.21	0.019*	0.03	0.38	-0.03	0.583	-0.13	0.08	0.24	0.041*	0.01	0.46	0.10	0.452	-0.15	0.34
Pos. Thalamic Radiation	0.17	0.432	-0.15	0.66	-0.11	0.089	-0.24	0.02	0.26	0.390	-0.24	0.90	0.09	0.568	-0.22	0.42
Association Fibers																
Cingulum	-0.13	0.542	-0.57	0.28	0.13	0.291	-0.11	0.38	-0.20	0.545	-0.84	0.42	-0.48	0.273	-1.34	0.23
External Capsule	0.19	0.222	-0.10	0.50	-0.01	0.938	-0.13	0.14	0.22	0.340	-0.22	0.69	-0.13	0.654	-0.70	0.40
Sagittal Stratum	0.30	0.071	-0.04	0.62	0.01	0.960	-0.23	0.26	0.30	0.151	-0.12	0.70	0.12	0.592	-0.38	0.47
Superior Fronto-occipital	0.04	0.779	-0.26	0.34	-0.05	0.713	-0.31	0.21	-0.06	0.768	-0.50	0.35	-0.11	0.584	-0.49	0.27
Superior Longitudinal	0.27	0.053	0.00	0.55	0.08	0.293	-0.08	0.23	0.31	0.103	-0.07	0.67	0.08	0.472	-0.15	0.29
**Non-verbal IQ**																
Average	0.50	0.001*	0.20	0.78	0.23	0.011*	0.05	0.39	0.57	0.001*	0.20	0.89	0.10	0.454	-0.17	0.35
Commissural Fibers																
Corpus Callosum	0.53	0.002*	0.19	0.86	0.19	0.115	-0.05	0.40	0.69	0.001*	0.26	1.09	0.28	0.105	-0.09	0.60
Fornix	0.49	0.017*	0.12	0.91	0.58	0.023*	0.10	1.08	0.57	0.016*	0.11	1.05	0.08	0.663	-0.25	0.43
Projection Fibers																
Cerebellar Peduncles	0.16	0.290	-0.14	0.46	0.33	0.006*	0.07	0.54	0.07	0.721	-0.31	0.46	-0.17	0.291	-0.48	0.15
Corona Radiata	0.63	<0.001*	0.28	0.97	0.22	0.104	-0.06	0.46	0.72	<0.001*	0.32	1.08	0.23	0.114	-0.07	0.50
Corticospinal Tract	0.11	0.343	-0.12	0.35	0.05	0.545	-0.11	0.21	0.12	0.530	-0.26	0.48	-0.16	0.351	-0.47	0.18
Internal Capsule	0.24	0.011*	0.05	0.42	0.19	0.018*	0.02	0.34	0.18	0.122	-0.05	0.41	-0.08	0.516	-0.30	0.15
Pos.Thalamic Radiation	0.28	0.170	-0.06	0.72	0.14	0.193	-0.07	0.35	0.27	0.314	-0.19	0.83	0.05	0.774	-0.27	0.36
Association Fibers																
Cingulum	-0.21	0.353	-0.64	0.24	0.06	0.738	-0.27	0.47	-0.22	0.465	-0.78	0.40	-0.35	0.144	-0.82	0.10
External Capsule	0.24	0.099	-0.04	0.55	0.06	0.688	-0.24	0.36	0.26	0.181	-0.11	0.65	-0.12	0.413	-0.41	0.18
Sagittal Stratum	0.46	0.013*	0.10	0.82	0.27	0.191	-0.13	0.67	0.40	0.096	-0.06	0.87	0.00	0.998	-0.43	0.46
Superior Fronto-occipital	0.21	0.280	-0.16	0.61	0.25	0.380	-0.29	0.82	0.08	0.774	-0.44	0.59	0.01	0.972	-0.49	0.57
Superior Longitudinal	0.31	0.042*	0.00	0.61	0.08	0.529	-0.16	0.33	0.39	0.049	-0.02	0.76	0.10	0.488	-0.19	0.39

This twin pair difference score analysis explores the putative genetic pathway underlying a pair of shared traits or phenotypes, which in this case are comprised of fractional anisotropy in different white matter tracts in the brain and intelligence quotient (IQ) scores from the Stanford-Binet, including full-scale, verbal, and non-verbal IQ. This analysis provides quantitative estimates of the Contribution to Pathway Covariance (CPC) from additive genetic factors. CPCs may be either positive or negative and may have the same or opposite signs acting to offset each other. Significant CPC at ***** $p < 0.05$.

Table S5. Contribution to pathway covariance for additive genetic effects on the shared traits between autism symptom severity and fractional anisotropy.

	All TWIN PAIRS				CONTROL PAIRS				ASD PAIRS				CONCORDANT ASD PAIRS			
**Total SRS**	Obs. Coef.	P>|z|	95% Conf. Interval		Obs. Coef.	P>|z|	95% Conf. Interval		Obs. Coef.	P>|z|	95% Conf. Interval		Obs. Coef.	P>|z|	95% Conf. Interval	
Average	-0.25	0.156	-0.57	0.13	0.03	0.118	-0.01	0.07	-0.33	0.167	-0.76	0.18	-0.07	0.825	-0.60	0.57
Commissural Fibers																
Corpus Callosum	-0.24	0.235	-0.61	0.18	0.01	0.578	-0.04	0.07	-0.36	0.186	-0.87	0.23	-0.17	0.612	-0.78	0.50
Fornix	0.22	0.311	-0.18	0.66	-0.04	0.449	-0.14	0.04	0.21	0.423	-0.30	0.76	0.84	0.013*	0.13	1.45
Projection Fibers																
Cerebellar Peduncles	-0.12	0.464	-0.44	0.21	0.03	0.389	-0.05	0.11	-0.36	0.137	-0.80	0.13	-0.41	0.056	-0.82	0.03
Corona Radiata	-0.27	0.196	-0.65	0.17	0.05	0.054	0.00	0.09	-0.27	0.311	-0.76	0.31	0.05	0.885	-0.62	0.79
Corticospinal Tract	-0.09	0.519	-0.35	0.19	-0.02	0.400	-0.08	0.03	-0.18	0.409	-0.58	0.27	-0.16	0.534	-0.65	0.34
Internal Capsule	-0.14	0.207	-0.34	0.08	0.03	0.253	-0.03	0.08	-0.13	0.401	-0.43	0.18	-0.13	0.521	-0.50	0.28
Pos.Thalamic Radiation	-0.18	0.365	-0.59	0.20	0.04	0.243	-0.03	0.10	-0.22	0.442	-0.78	0.36	0.11	0.708	-0.42	0.72
Association Fibers																
Cingulum	0.27	0.251	-0.19	0.74	0.05	0.103	-0.01	0.12	0.29	0.393	-0.37	0.93	0.70	0.109	-0.13	1.59
External Capsule	-0.44	0.015*	-0.79	-0.09	-0.05	0.109	-0.12	0.01	-0.61	0.011*	-1.06	-0.12	-0.25	0.325	-0.72	0.29
Sagittal Stratum	-0.35	0.075	-0.72	0.05	0.07	0.080	-0.01	0.16	-0.43	0.085	-0.92	0.07	-0.24	0.454	-0.82	0.44
Superior Fronto-occipital	0.28	0.238	-0.17	0.76	-0.09	0.061	-0.18	0.01	0.55	0.063	-0.02	1.15	0.99	0.025*	0.12	1.83
Superior Longitudinal	-0.14	0.425	-0.46	0.22	0.07	0.023*	0.01	0.13	-0.18	0.481	-0.65	0.34	-0.06	0.841	-0.61	0.57
**SCI**																
Average	-0.24	0.189	-0.57	0.15	0.03	0.136	-0.01	0.07	-0.32	0.195	-0.77	0.21	-0.08	0.809	-0.63	0.57
Commissural Fibers																
Corpus Callosum	-0.22	0.283	-0.60	0.21	0.01	0.793	-0.05	0.07	-0.34	0.235	-0.87	0.28	-0.17	0.614	-0.82	0.52
Fornix	0.23	0.294	-0.18	0.69	-0.06	0.247	-0.16	0.03	0.25	0.372	-0.27	0.81	0.86	0.012*	0.14	1.47
Projection Fibers																
Cerebellar Peduncles	-0.14	0.431	-0.47	0.21	0.05	0.145	-0.02	0.10	-0.39	0.124	-0.86	0.12	-0.48	0.033*	-0.91	-0.02
Corona Radiata	-0.25	0.247	-0.64	0.20	0.06	0.040*	0.00	0.11	-0.26	0.360	-0.76	0.35	0.06	0.870	-0.62	0.81
Corticospinal Tract	-0.07	0.623	-0.34	0.22	0.00	0.895	-0.05	0.05	-0.17	0.460	-0.58	0.30	-0.14	0.561	-0.63	0.35
Internal Capsule	-0.13	0.232	-0.34	0.09	0.03	0.219	-0.02	0.08	-0.13	0.396	-0.43	0.19	-0.15	0.475	-0.52	0.27
Pos. Thalamic Radiation	-0.18	0.368	-0.60	0.21	0.04	0.210	-0.03	0.11	-0.24	0.421	-0.81	0.35	0.09	0.750	-0.44	0.72
Association Fibers																
Cingulum	0.28	0.243	-0.19	0.76	0.05	0.187	-0.02	0.12	0.31	0.367	-0.37	0.97	0.69	0.126	-0.16	1.61
External Capsule	-0.43	0.018*	-0.79	-0.08	-0.05	0.094	-0.11	0.01	-0.61	0.013*	-1.07	-0.11	-0.26	0.326	-0.74	0.30
Sagittal Stratum	-0.34	0.091	-0.72	0.07	0.08	0.111	-0.02	0.17	-0.43	0.101	-0.94	0.09	-0.23	0.471	-0.83	0.45
Superior Fronto-occipital	0.28	0.238	-0.17	0.78	-0.10	0.066	-0.20	0.01	0.57	0.070	-0.03	1.20	1.00	0.027	0.11	1.86
Superior Longitudinal	-0.13	0.469	-0.47	0.25	0.07	0.030*	0.00	0.13	-0.17	0.515	-0.67	0.37	-0.04	0.903	-0.61	0.61
**RRB**																
Average	-0.25	0.120	-0.55	0.09	0.01	0.604	-0.04	0.07	-0.27	0.185	-0.64	0.17	0.07	0.773	-0.39	0.61
Commissural Fibers																
Corpus Callosum	-0.25	0.178	-0.59	0.14	0.03	0.321	-0.03	0.09	-0.34	0.153	-0.78	0.15	0.01	0.974	-0.50	0.58
Fornix	0.11	0.639	-0.35	0.57	-0.03	0.704	-0.19	0.12	0.16	0.524	-0.32	0.67	0.71	0.026*	0.07	1.31
Projection Fibers																
Cerebellar Peduncles	-0.10	0.528	-0.39	0.21	0.01	0.845	-0.10	0.16	-0.26	0.173	-0.63	0.13	-0.24	0.168	-0.56	0.11
Corona Radiata	-0.31	0.113	-0.66	0.10	0.01	0.777	-0.06	0.07	-0.24	0.299	-0.66	0.25	0.14	0.667	-0.46	0.79
Corticospinal Tract	-0.11	0.391	-0.35	0.14	-0.07	0.200	-0.18	0.03	-0.17	0.365	-0.53	0.21	-0.11	0.641	-0.58	0.35
Internal Capsule	-0.13	0.210	-0.33	0.08	0.01	0.794	-0.06	0.07	-0.06	0.671	-0.33	0.22	0.01	0.937	-0.32	0.35
Pos.Thalamic Radiation	-0.17	0.400	-0.57	0.22	-0.01	0.715	-0.08	0.06	-0.09	0.744	-0.63	0.47	0.25	0.360	-0.24	0.80
Association Fibers																
Cingulum	0.27	0.229	-0.16	0.70	0.06	0.120	-0.02	0.13	0.17	0.562	-0.41	0.76	0.66	0.066	-0.02	1.40
External Capsule	-0.38	0.023*	-0.70	-0.06	0.00	0.905	-0.08	0.07	-0.58	0.007*	-0.98	-0.13	-0.21	0.337	-0.62	0.26
Sagittal Stratum	-0.33	0.074	-0.69	0.04	0.02	0.490	-0.05	0.09	-0.35	0.128	-0.79	0.11	-0.12	0.680	-0.63	0.49
Superior Fronto-occipital	-0.13	0.398	-0.42	0.17	0.03	0.316	-0.03	0.09	-0.14	0.514	-0.55	0.29	-0.03	0.896	-0.50	0.47
Superior Longitudinal	0.18	0.457	-0.28	0.66	-0.06	0.522	-0.23	0.13	0.53	0.049*	0.02	1.07	0.92	0.021	0.12	1.66

This twin pair difference score analysis explores the putative genetic pathway underlying a pair of shared traits or phenotypes, which in this case are comprised of fractional anisotropy in different white matter tracts in the brain and autism-related symptom severity on the Social Responsiveness Scale (SRS), including Social Communication Impairments (SCI) and Restricted, Repetitive Behaviors (RRB). This analysis provides quantitative estimates of the Contribution to Pathway Covariance (CPC) from additive genetic factors. CPCs may be either positive or negative and may have the same or opposite signs acting to offset each other. Significant CPC at ***** $p < 0.05$.

Table S6. Contribution to pathway covariance for unique, unshared environmental effects on the shared traits between general cognitive abilities and fractional anisotropy

	All TWIN PAIRS				CONTROL PAIRS				ASD PAIRS				CONCORDANT ASD PAIRS			
**Full Scale IQ**	Obs. Coef.	P>|z|	95% Conf. Interval		Obs. Coef.	P>|z|	95% Conf. Interval		Obs. Coef.	P>|z|	95% Conf. Interval		Obs. Coef.	P>|z|	95%Conf. Interval	
Average	-0.010	0.521	-0.041	0.020	-0.009	0.389	-0.028	0.011	0.003	0.912	-0.055	0.060	0.007	0.846	-0.065	0.081
Commissural Fibers																
Corpus Callosum	-0.012	0.347	-0.037	0.012	-0.008	0.629	-0.039	0.026	0.005	0.859	-0.048	0.060	0.013	0.704	-0.056	0.083
Fornix	-0.012	0.534	-0.052	0.024	-0.017	0.364	-0.055	0.018	-0.003	0.907	-0.065	0.053	-0.004	0.924	-0.082	0.070
Projection Fibers																
Cerebellar Peduncles	-0.002	0.950	-0.065	0.055	-0.021	0.322	-0.067	0.016	0.009	0.866	-0.106	0.102	0.013	0.845	-0.125	0.127
Corona Radiata	-0.010	0.511	-0.039	0.019	0.000	0.978	-0.027	0.033	-0.006	0.844	-0.064	0.056	-0.004	0.920	-0.072	0.078
Corticospinal Tract	0.031	0.211	-0.013	0.083	-0.003	0.873	-0.032	0.031	0.072	0.074	-0.003	0.154	0.077	0.105	-0.014	0.166
Internal Capsule	-0.001	0.978	-0.042	0.042	0.002	0.845	-0.016	0.018	0.008	0.833	-0.063	0.082	0.006	0.906	-0.083	0.102
Pos.Thalamic Radiation	-0.012	0.540	-0.051	0.028	0.005	0.692	-0.021	0.031	-0.037	0.397	-0.118	0.054	-0.028	0.600	-0.128	0.085
Association Fibers																
Cingulum	-0.024	0.298	-0.070	0.019	-0.028	0.221	-0.074	0.016	0.008	0.819	-0.066	0.077	0.030	0.389	-0.041	0.097
External Capsule	-0.003	0.888	-0.047	0.037	-0.005	0.684	-0.033	0.020	0.007	0.854	-0.077	0.083	0.009	0.866	-0.100	0.108
Sagittal Stratum	-0.002	0.940	-0.057	0.057	-0.041	0.040*	-0.081	-0.002	0.037	0.425	-0.054	0.130	0.035	0.546	-0.080	0.146
Superior Fronto-occipital	-0.006	0.842	-0.061	0.053	0.004	0.829	-0.033	0.038	0.004	0.943	-0.097	0.112	0.001	0.987	-0.124	0.137
Superior Longitudinal	-0.007	0.687	-0.039	0.026	-0.011	0.567	-0.051	0.027	-0.001	0.970	-0.042	0.043	-0.004	0.874	-0.056	0.052
**Verbal IQ**																
Average	-0.021	0.268	-0.061	0.014	-0.013	0.202	-0.032	0.009	-0.005	0.870	-0.072	0.053	-0.003	0.937	-0.086	0.073
Commissural Fibers																
Corpus Callosum	-0.033	0.022*	-0.064	-0.006	-0.017	0.446	-0.063	0.026	-0.016	0.532	-0.068	0.035	-0.003	0.925	-0.069	0.060
Fornix	0.000	0.996	-0.051	0.046	0.017	0.500	-0.030	0.066	-0.012	0.716	-0.084	0.048	-0.017	0.680	-0.104	0.060
Projection Fibers																
Cerebellar Peduncles	0.001	0.983	-0.094	0.077	-0.013	0.567	-0.060	0.030	0.000	0.994	-0.150	0.113	-0.012	0.885	-0.192	0.123
Corona Radiata	-0.024	0.087	-0.052	0.004	-0.014	0.375	-0.041	0.021	-0.011	0.675	-0.062	0.045	0.002	0.942	-0.060	0.076
Corticospinal Tract	0.020	0.490	-0.032	0.080	0.019	0.506	-0.033	0.078	0.030	0.348	-0.032	0.092	0.058	0.074	-0.007	0.119
Internal Capsule	-0.007	0.785	-0.060	0.043	-0.002	0.853	-0.019	0.017	0.005	0.904	-0.077	0.083	-0.006	0.901	-0.107	0.093
Pos. Thalamic Radiation	0.012	0.580	-0.032	0.055	0.021	0.175	-0.010	0.051	-0.006	0.901	-0.103	0.085	-0.023	0.689	-0.138	0.089
Association Fibers																
Cingulum	-0.045	0.121	-0.105	0.009	-0.029	0.242	-0.079	0.017	-0.017	0.704	-0.109	0.062	0.018	0.621	-0.057	0.087
External Capsule	-0.015	0.611	-0.078	0.037	-0.006	0.681	-0.033	0.022	-0.012	0.800	-0.116	0.070	-0.018	0.769	-0.154	0.090
Sagittal Stratum	0.004	0.895	-0.053	0.070	-0.051	0.005*	-0.086	-0.014	0.060	0.186	-0.025	0.149	0.041	0.387	-0.049	0.135
Superior Fronto-occipital	-0.034	0.271	-0.099	0.024	0.002	0.899	-0.037	0.038	-0.041	0.394	-0.141	0.048	-0.046	0.458	-0.177	0.066
Superior Longitudinal	-0.005	0.779	-0.042	0.032	-0.021	0.321	-0.060	0.025	0.013	0.494	-0.023	0.053	0.013	0.601	-0.033	0.065
**Non-verbal IQ**																
Average	0.003	0.848	-0.026	0.031	-0.003	0.799	-0.027	0.018	0.013	0.642	-0.043	0.065	0.015	0.666	-0.054	0.083
Commissural Fibers																
Corpus Callosum	0.011	0.502	-0.019	0.043	0.001	0.936	-0.027	0.032	0.026	0.357	-0.032	0.081	0.027	0.445	-0.043	0.099
Fornix	-0.015	0.420	-0.051	0.020	-0.045	0.063	-0.097	-0.001	0.017	0.515	-0.037	0.068	0.010	0.782	-0.060	0.080
Projection Fibers																
Cerebellar Peduncles	-0.009	0.705	-0.057	0.037	-0.024	0.306	-0.074	0.019	0.004	0.930	-0.086	0.088	0.038	0.464	-0.072	0.133
Corona Radiata	0.000	0.995	-0.037	0.035	0.013	0.438	-0.019	0.045	-0.010	0.767	-0.079	0.057	-0.014	0.736	-0.091	0.071
Corticospinal Tract	0.034	0.221	-0.015	0.095	-0.024	0.028*	-0.044	-0.001	0.099	0.073	0.000	0.215	0.087	0.177	-0.030	0.212
Internal Capsule	0.009	0.620	-0.029	0.045	0.007	0.516	-0.015	0.025	0.015	0.667	-0.055	0.084	0.015	0.740	-0.074	0.106
Pos.Thalamic Radiation	-0.025	0.297	-0.072	0.021	-0.010	0.640	-0.053	0.028	-0.043	0.356	-0.130	0.054	-0.032	0.554	-0.133	0.083
Association Fibers																
Cingulum	0.011	0.657	-0.037	0.056	-0.030	0.291	-0.085	0.025	0.061	0.068	-0.007	0.123	0.038	0.276	-0.033	0.102
External Capsule	0.009	0.609	-0.026	0.045	-0.003	0.886	-0.040	0.032	0.025	0.496	-0.045	0.095	0.036	0.424	-0.055	0.124
Sagittal Stratum	-0.004	0.897	-0.060	0.051	-0.028	0.313	-0.083	0.024	0.021	0.676	-0.083	0.117	0.020	0.765	-0.119	0.147
Superior Fronto-occipital	0.018	0.559	-0.036	0.086	0.005	0.833	-0.041	0.047	0.038	0.548	-0.067	0.173	0.049	0.526	-0.076	0.210
Superior Longitudinal	-0.011	0.505	-0.045	0.021	0.001	0.980	-0.050	0.044	-0.024	0.307	-0.071	0.023	-0.025	0.407	-0.085	0.035

This twin pair difference score analysis explores the putative environmental pathway underlying a pair of shared traits or phenotypes, which in this case are comprised of fractional anisotropy in different white matter tracts in the brain and intelligence quotient (IQ) scores from the Stanford-Binet, including full-scale, verbal and non-verbal IQ. This analysis provides quantitative estimates of the Contribution to Pathway Covariance (CPC) from unique, unshared environmental factors. CPCs may be either positive or negative and may have the same or opposite signs acting to offset each other. Significant CPC at ***** $p < 0.05$.

Table S7. Contribution to pathway covariance for unique, unshared environmental effects on the shared traits between autism symptom severity and fractional anisotropy.

	All TWIN PAIRS				CONTROL PAIRS				ASD PAIRS				CONCORDANT ASD PAIRS			
**Total SRS**	Obs. Coef.	P>|z|	95% Conf. Interval		Obs. Coef.	P>|z|	95% Conf. Interval		Obs. Coef.	P>|z|	95% Conf. Interval		Obs. Coef.	P>|z|	95%Conf. Interval	
Average	0.040	0.156	-0.013	0.096	-0.005	0.278	-0.014	0.004	0.061	0.210	-0.031	0.158	0.078	0.193	-0.038	0.193
Commissural Fibers																
Corpus Callosum	0.037	0.267	-0.022	0.107	-0.006	0.420	-0.022	0.010	0.044	0.450	-0.062	0.166	0.062	0.390	-0.071	0.214
Fornix	-0.041	0.379	-0.141	0.044	0.029	0.171	-0.003	0.074	-0.130	0.049*	-0.261	0.000	-0.104	0.212	-0.275	0.045
Projection Fibers																
Cerebellar Peduncles	0.084	0.049*	0.004	0.168	-0.013	0.269	-0.037	0.010	0.203	0.005*	0.059	0.340	0.209	0.016*	0.036	0.375
Corona Radiata	0.030	0.377	-0.031	0.101	-0.003	0.739	-0.022	0.012	0.036	0.512	-0.066	0.148	0.049	0.477	-0.083	0.186
Corticospinal Tract	-0.033	0.259	-0.094	0.021	0.001	0.942	-0.018	0.019	-0.065	0.239	-0.176	0.042	-0.067	0.287	-0.189	0.058
Internal Capsule	0.019	0.490	-0.034	0.072	-0.003	0.576	-0.014	0.010	0.023	0.613	-0.067	0.111	0.045	0.412	-0.068	0.149
Pos.Thalamic Radiation	-0.002	0.960	-0.100	0.077	-0.001	0.877	-0.014	0.013	0.001	0.992	-0.190	0.164	-0.016	0.861	-0.211	0.141
Association Fibers																
Cingulum	0.020	0.626	-0.065	0.102	-0.014	0.196	-0.035	0.009	0.012	0.859	-0.125	0.150	0.077	0.210	-0.040	0.203
External Capsule	0.084	0.007*	0.024	0.145	0.007	0.297	-0.007	0.021	0.159	0.001*	0.053	0.250	0.176	0.004*	0.045	0.285
Sagittal Stratum	0.011	0.733	-0.056	0.076	-0.002	0.853	-0.022	0.017	0.036	0.515	-0.076	0.145	0.054	0.392	-0.071	0.174
Superior Fronto-occipital	0.008	0.894	-0.117	0.113	0.031	0.037*	0.004	0.060	-0.052	0.577	-0.245	0.112	-0.108	0.350	-0.333	0.110
Superior Longitudinal	0.020	0.484	-0.030	0.083	-0.017	0.059	-0.036	0.000	0.060	0.298	-0.050	0.177	0.060	0.375	-0.064	0.202
**SCI**																
Average	0.046	0.118	-0.009	0.105	-0.006	0.273	-0.016	0.003	0.074	0.145	-0.022	0.177	0.095	0.126	-0.027	0.215
Commissural Fibers																
Corpus Callosum	0.041	0.243	-0.021	0.115	-0.008	0.376	-0.025	0.009	0.053	0.386	-0.058	0.180	0.073	0.327	-0.064	0.231
Fornix	-0.036	0.442	-0.135	0.050	0.029	0.162	-0.003	0.075	-0.120	0.065	-0.248	0.010	-0.090	0.273	-0.257	0.058
Projection Fibers																
Cerebellar Peduncles	0.095	0.032*	0.013	0.185	-0.007	0.399	-0.021	0.011	0.222	0.005*	0.066	0.372	0.229	0.015*	0.043	0.412
Corona Radiata	0.033	0.346	-0.030	0.106	-0.007	0.522	-0.029	0.009	0.046	0.414	-0.061	0.162	0.059	0.397	-0.076	0.199
Corticospinal Tract	-0.034	0.280	-0.099	0.023	-0.005	0.601	-0.021	0.013	-0.060	0.302	-0.177	0.052	-0.063	0.329	-0.191	0.064
Internal Capsule	0.025	0.376	-0.029	0.080	-0.003	0.586	-0.014	0.010	0.036	0.444	-0.057	0.127	0.062	0.267	-0.054	0.168
Pos. Thalamic Radiation	0.002	0.960	-0.094	0.082	-0.002	0.789	-0.015	0.013	0.012	0.896	-0.178	0.177	0.006	0.947	-0.188	0.162
Association Fibers																
Cingulum	0.021	0.632	-0.069	0.104	-0.015	0.209	-0.039	0.009	0.014	0.854	-0.136	0.159	0.087	0.174	-0.036	0.216
External Capsule	0.091	0.006*	0.028	0.157	0.012	0.054	0.000	0.025	0.169	0.001*	0.058	0.265	0.188	0.004*	0.046	0.305
Sagittal Stratum	0.016	0.648	-0.053	0.083	-0.003	0.771	-0.025	0.018	0.048	0.423	-0.074	0.160	0.071	0.268	-0.058	0.193
Superior Fronto-occipital	0.008	0.893	-0.118	0.116	0.028	0.065	0.000	0.058	-0.049	0.602	-0.243	0.117	-0.106	0.361	-0.331	0.116
Superior Longitudinal	0.024	0.422	-0.028	0.088	-0.016	0.077	-0.034	0.001	0.066	0.268	-0.049	0.185	0.065	0.344	-0.063	0.209
**RRB**																
Average	0.013	0.583	-0.033	0.060	-0.007	0.288	-0.020	0.004	0.006	0.885	-0.074	0.087	0.008	0.875	-0.092	0.113
Commissural Fibers																
Corpus Callosum	0.013	0.644	-0.041	0.072	-0.011	0.125	-0.024	0.004	-0.001	0.988	-0.097	0.097	0.005	0.941	-0.119	0.135
Fornix	-0.060	0.223	-0.167	0.025	0.019	0.397	-0.019	0.068	-0.161	0.036*	-0.314	-0.019	-0.151	0.120	-0.345	0.017
Projection Fibers																
Cerebellar Peduncles	0.044	0.236	-0.033	0.113	-0.033	0.267	-0.097	0.016	0.140	0.009*	0.030	0.242	0.146	0.020*	0.015	0.263
Corona Radiata	0.015	0.632	-0.044	0.077	0.003	0.730	-0.010	0.019	-0.002	0.968	-0.096	0.101	0.009	0.890	-0.120	0.145
Corticospinal Tract	-0.041	0.179	-0.101	0.018	0.014	0.567	-0.029	0.066	-0.097	0.077	-0.203	0.013	-0.096	0.128	-0.221	0.029
Internal Capsule	-0.006	0.818	-0.059	0.044	-0.005	0.464	-0.019	0.008	-0.028	0.542	-0.117	0.062	-0.021	0.707	-0.133	0.090
Pos.Thalamic Radiation	-0.018	0.721	-0.130	0.070	0.007	0.526	-0.014	0.027	-0.043	0.663	-0.250	0.134	-0.096	0.346	-0.310	0.077
Association Fibers																
Cingulum	0.013	0.727	-0.058	0.092	-0.018	0.268	-0.051	0.014	0.003	0.954	-0.102	0.121	0.034	0.566	-0.076	0.158
External Capsule	0.059	0.020*	0.009	0.107	-0.008	0.589	-0.038	0.017	0.121	0.006*	0.023	0.197	0.132	0.007*	0.022	0.217
Sagittal Stratum	-0.006	0.866	-0.079	0.061	-0.003	0.774	-0.020	0.016	-0.001	0.984	-0.114	0.110	-0.002	0.977	-0.138	0.133
Superior Fronto-occipital	-0.003	0.964	-0.134	0.098	0.035	0.020*	0.006	0.064	-0.078	0.448	-0.286	0.101	-0.131	0.295	-0.373	0.101
Superior Longitudinal	0.012	0.683	-0.037	0.080	-0.011	0.306	-0.032	0.010	0.035	0.535	-0.068	0.154	0.036	0.598	-0.083	0.181

This twin pair difference score analysis explores the putative environmental pathway underlying a pair of shared traits or phenotypes, which in this case are comprised of fractional anisotropy in different white matter tracts in the brain and intelligence quotient (IQ) scores from the Stanford-Binet, including full-scale, verbal and non-verbal IQ. This analysis provides quantitative estimates of the Contribution to Pathway Covariance (CPC) from unique, unshared environmental factors. CPCs may be either positive or negative and may have the same or opposite signs acting to offset each other. Significant CPC at ***** $p < 0.05$.

References

1. Lord C, Rutter M, DiLavore P, Risi S, Gotham K, Bishop S. *Autism Diagnostic Observation Schedule–2nd edition (ADOS-2)*. Western Psychological Corporation; 2012.

2. Lord C, Rutter M, Le Couteur A. Autism Diagnostic Interview-Revised: a revised version of a diagnostic interview for caregivers of individuals with possible pervasive developmental disorders. *J Autism Dev Disord*. Oct 1994;24(5):659-85. doi:10.1007/bf02172145

3. Constantino JN, Gruber CP. *Social responsiveness scale (SRS)*. Western Psychological Services Los Angeles, CA; 2007.

4. Frazier TW, Ratliff KR, Gruber C, Zhang Y, Law PA, Constantino JN. Confirmatory factor analytic structure and measurement invariance of quantitative autistic traits measured by the Social Responsiveness Scale-2. *Autism*. 2014/01/01 2013;18(1):31-44. doi:10.1177/1362361313500382

5. Roid GH. *Stanford-Binet intelligence scales*. Riverside Publishing Itasca, IL; 2003.

6. Akshoomoff N. Use of the Mullen Scales of Early Learning for the assessment of young children with Autism Spectrum Disorders. *Child Neuropsychol*. 2006;12(4-5):269-277. doi:10.1080/09297040500473714

7. Mullen EM. *Mullen scales of early learning*. American Guidance Service; 1995.

8. Hus V, Bishop S, Gotham K, Huerta M, Lord C. Factors influencing scores on the social responsiveness scale. *Journal of child psychology and psychiatry, and allied disciplines*. 2013;54(2):216-224. doi:10.1111/j.1469-7610.2012.02589.x

9. Constantino JN, Frazier TW. Commentary: The observed association between autistic severity measured by the social responsiveness scale (SRS) and general psychopathology--a response to Hus et al.(2013). *Journal of child psychology and psychiatry, and allied disciplines*. 2013;54(6):695-697. doi:10.1111/jcpp.12064

10. Lundström S, Chang Z, Kerekes N, et al. Autistic-like traits and their association with mental health problems in two nationwide twin cohorts of children and adults. *Psychological Medicine*. 2011;41(11):2423-2433. doi:10.1017/S0033291711000377

11. Clarke TK, Lupton MK, Fernandez-Pujals AM, et al. Common polygenic risk for autism spectrum disorder (ASD) is associated with cognitive ability in the general population. *Molecular Psychiatry*. 2016/03/01 2016;21(3):419-425. doi:10.1038/mp.2015.12

12. Merboldt K-D, Hänicke W, Frahm J. Diffusion imaging using stimulated echoes. *Magnetic Resonance in Medicine*. 1991/06/01 1991;19(2):233-239. doi:https://doi.org/10.1002/mrm.1910190208

13. *NiftyReg*. 2010. https://iris.ucl.ac.uk/iris/publication/326396/1

14. Smith SM, Jenkinson M, Johansen-Berg H, et al. Tract-based spatial statistics: Voxelwise analysis of multi-subject diffusion data. *NeuroImage*. 2006/07/15/ 2006;31(4):1487-1505. doi:https://doi.org/10.1016/j.neuroimage.2006.02.024

15. Bach M, Laun FB, Leemans A, et al. Methodological considerations on tract-based spatial statistics (TBSS). *Neuroimage*. 2014;100:358-369.

16. Smith SM, Jenkinson M, Woolrich MW, et al. Advances in functional and structural MR image analysis and implementation as FSL. *Neuroimage*. 2004;23(Supplement 1):S208-S219.

17. Mori S, Oishi K, Jiang H, et al. Stereotaxic white matter atlas based on diffusion tensor imaging in an ICBM template. *NeuroImage*. 2008/04/01/ 2008;40(2):570-582. doi:https://doi.org/10.1016/j.neuroimage.2007.12.035

18. Jahanshad N, Kochunov PV, Sprooten E, et al. Multi-site genetic analysis of diffusion images and voxelwise heritability analysis: A pilot project of the ENIGMA–DTI working group. *NeuroImage*. 2013/11/01/ 2013;81:455-469. doi:https://doi.org/10.1016/j.neuroimage.2013.04.061

19. Hegarty JP, Pegoraro LFL, Lazzeroni LC, et al. Genetic and environmental influences on structural brain measures in twins with autism spectrum disorder. *Molecular Psychiatry*. 2019/01/18 2019;doi:10.1038/s41380-018-0330-z

20. Hoaglin DC, Iglewicz B, Tukey JW. Performance of some resistant rules for outlier labeling. *Journal of the American Statistical Association*. 1986;81(396):991-999.

21. DeFries JC, Fulker DW. Multiple regression analysis of twin data. *Behavior Genetics*. 1985/09/01 1985;15(5):467-473. doi:10.1007/BF01066239

22. Lazzeroni LC, Ray A. A Generalized Defries–Fulker Regression Framework for the Analysis of Twin Data. *Behavior Genetics*. 2013/01/01 2013;43(1):85-96. doi:10.1007/s10519-012-9573-7

23. Pike A, Reiss D, Hetherington EM, Plomin R. Using MZ differences in the search for nonshared environmental effects. *Journal of Child Psychology and Psychiatry*. 1996;37(6):695-704.

24. Hegarty Ii JP, Lazzeroni LC, Raman MM, et al. Genetic and environmental influences on corticostriatal circuits in twins with autism. *Journal of psychiatry & neuroscience : JPN*. 2020;45(3):188-197. doi:10.1503/jpn.190030

25. Hegarty JP, II, Lazzeroni LC, Raman MM, et al. Genetic and Environmental Influences on Lobar Brain Structures in Twins With Autism. *Cerebral Cortex*. 2020;30(3):1946-1956. doi:10.1093/cercor/bhz215

26. Asbury K, Dunn JF, Pike A, Plomin R. Nonshared Environmental Influences on Individual Differences in Early Behavioral Development: A Monozygotic Twin Differences Study. *Child Development*. 2003/05/01 2003;74(3):933-943. doi:https://doi.org/10.1111/1467-8624.00577

27. Turkheimer E, Waldron M. Nonshared environment: a theoretical, methodological, and quantitative review. *Psychological bulletin*. 2000;126(1):78.

28. von Stumm S, Plomin R. Monozygotic twin differences in school performance are stable and systematic. *Developmental Science*. 2018/11/01 2018;21(6):e12694. doi:https://doi.org/10.1111/desc.12694

29. SAS Institute Inc. 2014. SAS/STAT®13.2 User's Guide. SAS Institute Inc. Cary, NC.

30. Benjamini Y, Hochberg Y. Controlling the false discovery rate: a practical and powerful approach to multiple testing. *Journal of the Royal Statistical Society*. 1995;57(1):289-300.
